# Regulatory Role of Anti-Sigma Factor RsbW in Clostridioides difficile Stress Response, Persistence, and Infection

**DOI:** 10.1128/jb.00466-22

**Published:** 2023-04-26

**Authors:** Jeffrey K. J. Cheng, Tanja Đapa, Ivan Y. L. Chan, Thomas O. MacCreath, Ross Slater, Meera Unnikrishnan

**Affiliations:** a Division of Biomedical Sciences, Warwick Medical School, University of Warwick, Coventry, United Kingdom; b Instituto Gulbenkian de Ciência, Oeiras, Portugal; Ohio State University

**Keywords:** *Clostridioides difficile*, anti-sigma factor RsbW, regulation, sigma factor B, stress responses

## Abstract

The anaerobic pathogen Clostridioides difficile, which is a primary cause of antibiotic-associated diarrhea, faces a variety of stresses in the environment and in the mammalian gut. To cope with these stresses, alternative sigma factor B (σ^B^) is employed to modulate gene transcription, and σ^B^ is regulated by an anti-sigma factor, RsbW. To understand the role of RsbW in C. difficile physiology, a *rsbW* mutant (Δ*rsbW*), in which σ^B^ is assumed to be “always on,” was generated. Δ*rsbW* did not show fitness defects in the absence of stress but tolerated acidic environments and detoxified reactive oxygen and nitrogen species better compared to the parental strain. Δ*rsbW* was defective in spore and biofilm formation, but it displayed increased adhesion to human gut epithelia and was less virulent in a Galleria mellonella infection model. A transcriptomic analysis to understand the unique phenotype of Δ*rsbW* showed changes in expression of genes associated with stress responses, virulence, sporulation, phage, and several σ^B^-controlled regulators, including the pleiotropic regulator sinRR′. While these profiles were distinct to Δ*rsbW*, changes in some σ^B^-controlled stress-associated genes were similar to those reported in the absence of σ^B^. Further analysis of Δ*rsbW* showed unexpected lower intracellular levels of σ^B^, suggesting an additional post-translational control mechanism for σ^B^ in the absence of stress. Our study provides insight into the regulatory role of RsbW and the complexity of regulatory networks mediating stress responses in C. difficile.

**IMPORTANCE** Pathogens like Clostridioides difficile face a range of stresses in the environment and within the host. Alternative transcriptional factors like sigma factor B (σ^B^) enable the bacterium to respond quickly to different stresses. Anti-sigma factors like RsbW control sigma factors and therefore the activation of genes via these pathways. Some of these transcriptional control systems provide C. difficile with the ability to tolerate and detoxify harmful compounds. Here, we investigate the role of RsbW in C. difficile physiology. We demonstrate distinctive phenotypes for a *rsbW* mutant in growth, persistence, and virulence and suggest alternate σ^B^ control mechanisms in C. difficile. Understanding C. difficile responses to external stress is key to designing better strategies to combat this highly resilient bacterial pathogen.

## INTRODUCTION

Clostridioides difficile is a Gram-positive, spore-forming, anaerobic bacterium found ubiquitously in nature and nosocomial environments. Transmission primarily occurs through the ingestion of spores via the fecal-oral route in health care and community settings. C. difficile can proliferate and induce symptoms of varying severity when the normal microbiota in the gastrointestinal (GI) tract is disrupted, usually as a result of antibiotic treatment. C. difficile infection (CDI) is typically associated with severe diarrhea and pseudomembranous colitis (1, 2). A greater awareness of CDI and antibiotic stewardship have reduced cases of health care-associated infections and in-hospital deaths, but recurrent infections remain a serious problem (3). The high rates of repeated episodes and associated increased mortality (4, 5) place a heavy cost burden on health care systems worldwide. One possible reason for high rates of recurrence is the ability of C. difficile to persist in extreme environmental conditions, in both hospital and community environments and within the host gut.

C. difficile spores are known to resist a range of environmental stresses, including human stomach acid (6, 7); however, upon germination, the vegetative cells are also exposed to a myriad of host-derived stressors. Each of the GI tract compartments, like the duodenum, jejunum, and ileum, present C. difficile with various levels of oxygen concentrations, pH, osmolarity, immune responses, and interspecies competition (8, 9). Like many other *Bacillota*, C. difficile possesses alternative sigma factors to enable it to respond to such external stimuli, one example being sigma factor B (σ^B^) (10). By changing the transcriptional target of the RNA polymerase, the bacterium can express a plethora of general stress response genes to ensure its adaptation and survival.

σ^B^ has been best characterized in Bacillus subtilis, in which seven other different proteins are involved in the activation of σ^B^ through the partner-switching complex, phosphatases, and a transducer stressosome complex (11–15). The number of alternative σ factors and Rsb proteins appear to differ between species, suggesting that this diversity can be associated with the variety of stresses encountered in its life cycle (16). In C. difficile, only the partner-switching complex and a protein phosphatase 2C (PP2C)-type phosphatase have been uncovered (17, 18). The genes encoding the post-translational partner-switching modules (RsbV and RsbW) are always in the σ^B^ operon and appear to be universally conserved in all strains expressing σ^B^ (Fig. S1A). In this post-translational paradigm, σ^B^ can exist in two states: unbound and bound. During normal growth in the absence of stress, anti-sigma factor RsbW sequesters σ^B^ to prevent unnecessary transcription (11, 19, 20). Only through the dephosphorylation of anti-anti-sigma factor RsbV can RsbW preferentially release σ^B^ in favor of binding RsbV (21). This event is mediated by a serine/threonine PP2C, RsbZ, and subsequently controls σ^B^ activation (18). The balance is restored through the additional activity of RsbW; acting as a kinase, it phosphorylates RsbV and returns to restraining σ^B^ (Fig. S1B) (22).

The roles of σ^B^ and associated Rsb proteins have been studied recently in C. difficile (17, 18, 23, 24). σ^B^ has been shown to be involved primarily in the stress responses that mediate detoxification of reactive oxidative species (ROS) and reactive nitrogen species (RNS) (17, 18, 23). σ^B^ has also been associated with tolerance to low oxygen, protection from acidic pH, DNA repair, tellurite, and thiol resistance (17, 18). Furthermore, σ^B^ mutants displayed increased sporulation frequency and enhanced colonization in a murine infection model (17).

Unlike σ^B^, the role of the anti-sigma factor RsbW during bacterial growth or under conditions of stress has been understudied. One of the reasons for this is that in many species, σ^B^ is autoregulated, causing uncontrolled transcription in the absence of RsbW, which results in a deleterious fitness phenotype, as shown in Bacillus (11). In C. difficile, it was presumed that a deletion mutant would be toxic (20, 25), and only an artificial strain overexpressing RsbW has been examined (18). However, the C. difficile σ^B^ operon appears to be controlled only by housekeeping σ^A^, unlike in other species, as a σ^B^ promoter has not been identified immediately upstream of the operon (26–28). This suggests that σ^B^ (*sigB*) gene transcription would be unaltered in the absence of RsbW, due to the missing feedback loop.

In this study, we created a *rsbW* deletion mutant in C. difficile R20291 to explore the regulatory roles of RsbW. In this strain, the lack of a partner-switching mechanism is expected to result in unbound σ^B^ and hence a “constitutive” expression of σ^B^. Here, we demonstrate the lack of a fitness defect in the Δ*rsbW* mutant in rich growth medium and increased survival in a variety of stress-inducing conditions. The absence of RsbW also affected C. difficile cell adhesion and virulence. Interestingly, an RNA-sequencing (RNA-Seq) analysis revealed that transcriptional changes were distinct yet unexpectedly similar to those previously reported for a σ^B^ mutant (17). Examination of relative intracellular concentrations of σ^B^ revealed a decreased level of σ^B^, indicating post-translational control. The results from this study demonstrate the role of RsbW in modulating stress responses and virulence and suggest alternative control mechanisms for σ^B^.

## RESULTS

### Deficiency in RsbW and RsbW overexpression does not cause a fitness defect *in vitro*.

A defective RsbW protein is thought to negatively affect the overall fitness of the bacterium as previously seen in B. subtilis (20, 25), as the presence of unbound σ^B^ could lead to unnecessary transcription and subsequent cellular toxicity. An in-frame *rsbW* deletion mutant (Δ*rsbW*) was constructed as described under Materials and Methods, such that the start codon of σ^B^ was maintained. To assess overall bacterial fitness, growth of the WT, Δ*rsbW*, and a complemented strain (Δ*rsbW*+*rsbW*), in which the *rsbW* gene was expressed episomally, was monitored in three different growth media: brain-heart infusion (BHI) with 0.5% (wt/vol) yeast extract and 0.1% (wt/vol) l-cysteine (BHI-S) medium (Fig. S2A), tryptone yeast (TY) medium (Fig. S2B), and Dulbecco’s minimal essential medium with 10% fetal bovine serum (DMEM-10) (Fig. S2C). Over the course of 12 h, only modest growth differences were observed between the three strains in the different media, and the strains had similar mean generation times (Fig. S2D). This suggests that the absence of RsbW does not cause a significant fitness defect in C. difficile when grown in rich growth medium in the absence of stress.

To confirm this and further assess effects of anhydrotetracycline (aTc)-mediated overexpression of RsbW, the WT, Δ*rsbW*, Δ*rsbW* with control plasmid pRPF185, or pRPF185+*rsbW* (complemented) strains were serially diluted onto BHI-S agar supplemented with/without thiamphenicol and aTc (Fig. S3A) (23). Δ*rsbW* + rsbW appeared to have a slight growth defect in comparison to the WT upon aTc induction (Fig. S3B), although this difference was not statistically significant (Fig. S3C). However, the Δ*rsbW* vector control and Δ*rsbW* also displayed similar decreases in growth (Fig. S3C), indicating that the defect was an effect of aTc rather than overexpression of RsbW. Thus, our data indicate that the absence of or increased expression of RsbW did not affect bacterial fitness under nonstressed conditions.

### RsbW affects C. difficile responses to stress.

**FIG 1:**
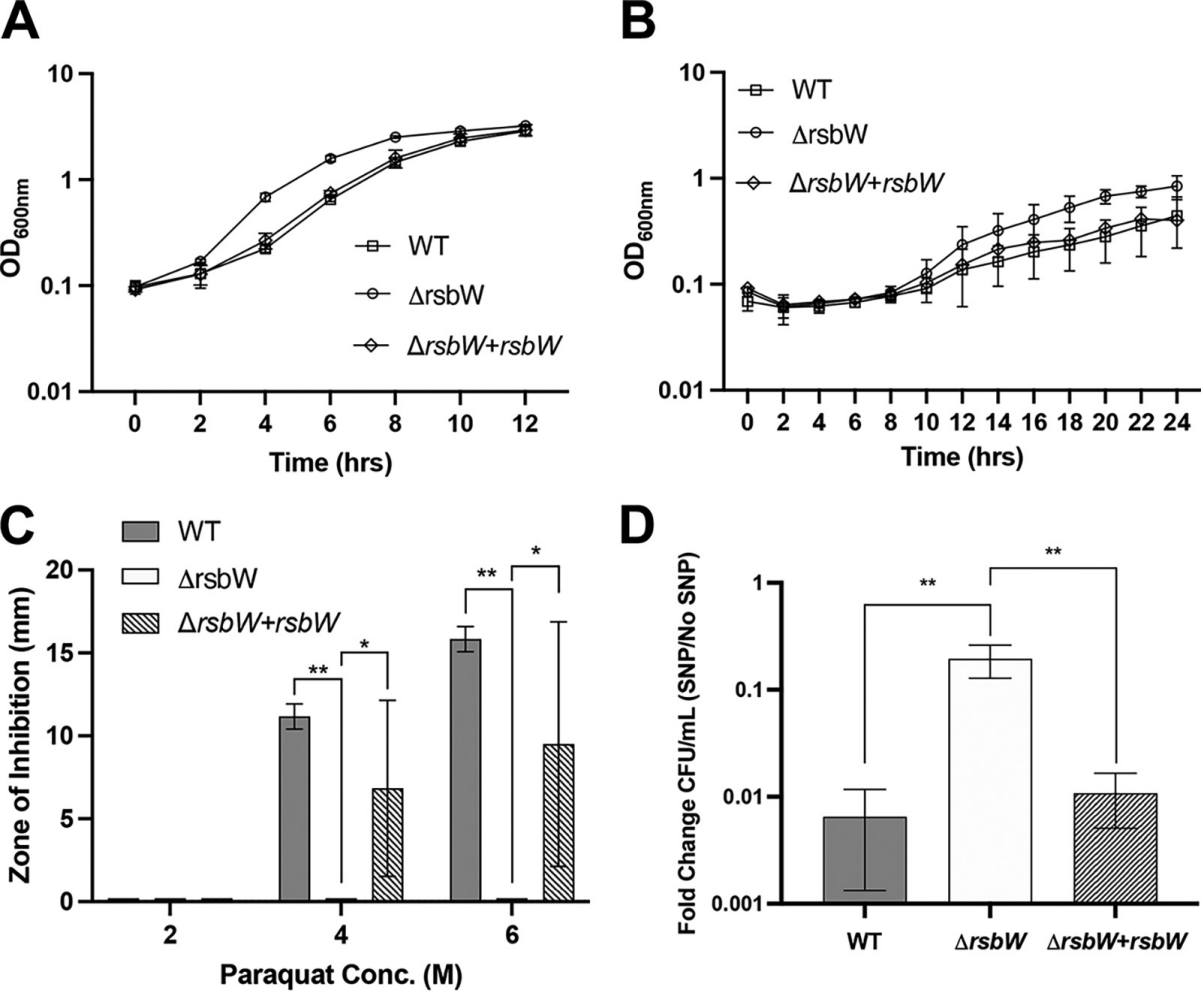
Tolerance of external stresses by Δ*rsbW.* (A, B) Growth of wild-type (WT), Δ*rsbW*, and complemented strain Δ*rsbW+rsbW* as measured by optical density at 600 nm (OD_600nm_) was compared in tryptone yeast medium in the presence of 1% oxygen (A) and brain-heart infusion (BHI) with 0.5% (wt/vol) yeast extract and 0.1% (wt/vol) l-cysteine (BHI-S) medium adjusted to pH 5 (B). (C) The zones of inhibition on BHI plates supplemented with 4 and 6 M paraquat were compared for WT, Δ*rsbW*, and Δ*rsbW+rsbW.* (D) Colony counts for WT, Δ*rsbW*, and Δ*rsbW+rsbW* on BHI-S agar supplemented with and without 1.5 mM sodium nitroprusside (*N* = 3, with 3 technical replicates/experiment). Error bars indicate standard deviation (SD). Significant differences are indicated by asterisks: *, *P* < 0.05; **, *P* < 0.01 as determined by Mann-Whitney U test.

The role of σ^B^ in C. difficile stress response has been characterized (17, 18, 23, 29); however, the contribution of RsbW has not been investigated. We first examined the effects of common environmental stresses to which C. difficile is likely exposed in the gut, such as oxygen and low pH. When C. difficile was grown in soft agar tubes in an aerobic environment as previously described by Kint et al. (17), only a small difference in the zones of growth was observed between the WT and *rsbW* mutant (Fig. S4A and S4B). However, when the strains were cultured in liquid broth with 1% oxygen (Fig. 1A), Δ*rsbW* had a higher mean generation time of 1.68 compared to the WT (2.05) and the complemented strain (1.93), indicated by the sharp increase in OD_600nm_ at 2 h (*P* = 0.013). When strains were cultured in acidic liquid growth medium at pH 5 (Fig. 1B), Δ*rsbW* showed an increased growth compared to the WT and complemented strains from 10 h onwards; however, the differences in mean generation time (WT, 6.35 and Δ*rsbW*, 4.38) were not statistically significant (Table S3). No clear differences were observed between the strains when cultured in growth media at pH 4, pH 6, and pH 7 (Fig. S5A; Table S3). Enumeration of CFU from these conditions further confirmed better survival of Δ*rsbW* at pH 5.0 compared to the WT (Fig. S5B).

Reactive oxygen species (ROS) and reactive nitrogen species (RNS) produced by immune cells are a key response to bacterial killing during infection. During CDI, neutrophils and macrophages are recruited to the site of colonization releasing ROS and RNS (30, 31). σ^B^ has been implicated in detoxification of these through upregulation of genes such as reverse-ruberythrins, NADH-rubredoxin, NO, and nitro-reductases (17, 23, 29). To assess the ability of the Δ*rsbW* to detoxify ROS and RNS, detoxification of H_2_O_2_ and superoxide anion O_2_^−^ (from paraquat) was assessed as described previously (17, 18, 23). Bacterial lawns of standardized overnight cultures were subjected to oxidative reagents dispensed onto sterile diffusion disks. Both WT and mutant bacterial lawns showed similar zones of inhibition with 1 M H_2_O_2_ (Fig. S6A) and no inhibition with 2 M paraquat (Fig. 1C). However, when exposed to higher concentrations, no zone of inhibition was visible for the Δ*rsbW* grown in the presence of 4 and 6 M paraquat (Fig. 1C) compared to the WT (x¯ 11.2 and 15.8 mm, respectively) and complemented strain (x¯ 6.8 and 9.5 mm, respectively), indicating that the mutant strain was able to effectively detoxify the toxic compound (*P* = 0.003). H_2_O_2_ treatment at 2 and 4 M produced no difference in bacterial killing, while concentrations of 9.8 M exhibited a slightly decreased zone of inhibition for Δ*rsbW* (~6%) compared to the WT (Fig. S6A).

We next tested sensitivity to RNS (17, 18). Standardized bacterial cultures were spot diluted on agar plates supplemented with sodium nitroprusside (SNP), a nitric-oxide donor compound, and bacterial numbers were enumerated. Δ*rsbW* growth was not affected at lower concentrations of 0.2 and 0.5 mM SNP (Fig. S6B). At 1 mM SNP, differences were seen between the WT (and plasmid controls) and Δ*rsbW* (Fig. S6B), although these differences were not statistically significant. However, a significant difference in the mean fold change in CFU/mL was observed in bacterial survival with SNP at 1.5 mM (Fig. 1D) between Δ*rsbW* (0.1951) and the WT (0.0065), indicating that the Δ*rsbW* can reduce nitric oxide (NO) more efficiently than the WT (*P* = 0.002). Thus, these results indicate that the *rsbW* mutant exhibits better survival than the WT strain under conditions of high levels of oxidative and nitrosative stress.

### RsbW controls sporulation.

**FIG 2:**
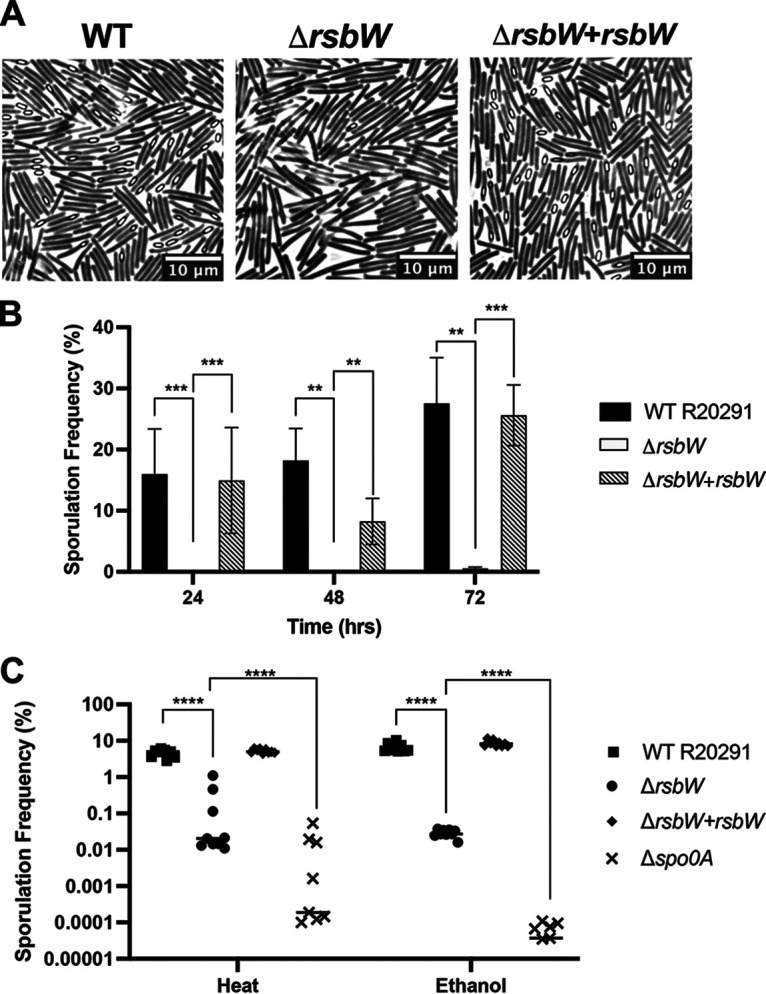
Δ*rsbW* displays severe defects in sporulation. C. difficile strains WT, Δ*rsbW*, and Δ*rsbW*+*rsbW* were grown on 70:30 sporulation medium. (A) Representative phase-contrast microscopy images were taken after 24 h (*N* = 3, with 5 representative fields/experiment). (B) Visible spores were enumerated from the microscopy images and compared with total number of cells. (C) Bacteria cultured on 70:30 sporulation medium were diluted to OD_600nm_ 1.0 and subjected to heat and ethanol treatment. Germination frequency was calculated from colony counts of cultures before and after treatment, on brain-heart infusion with 0.5% (wt/vol) yeast extract and 0.1% (wt/vol) l-cysteine (BHI-S) ± 0.1% taurocholate (*N* = 3, with 3 technical replicates/experiment). Error bars indicate SD. Significant differences are denoted with asterisks: **, *P* < 0.01; ***, *P* < 0.001; ****, *P* < 0.0001 as determined Student’s *t* test or Mann-Whitney U test.

Production of spores is a key mechanism by which C. difficile evades unfavorable conditions. Sporulation in C. difficile is controlled by many regulators and four sporulation-associated σ factors (32, 33). Sporulation is negatively associated with σ^B^; a 10-fold increase in sporulation rate was reported in a σ^B^ mutant, while the germination efficiency was unaffected (17). In sporulation assays, where strains were grown on 70:30 sporulation media, a dramatic decrease in Δ*rsbW* sporulation was observed when examined under phase-contrast microscopy (Fig. 2A and B), suggesting an “always on” state for σ^B^ in Δ*rsbW*. Total spores to bacterial cells per microscope image were enumerated across 72 h, Δ*rsbW* had a sporulation frequency of less than 1%, while the WT and complemented strain showed rates of ~15% to 37%. To assess spore viability, the number of spores produced were quantified by measuring CFU in the presence of germination agent, sodium taurocholate, after heat or ethanol treatment, using a *spo0A* mutant as the negative control (Fig. 2C). More than a 100-fold difference was observed between strains, with a germination frequency of ~16% for the WT compared to ~0.05% for Δ*rsbW* in either treatment condition. The ability to form spores was restored in the *rsbW* complemented strain. Thus, the absence of RsbW negatively affects sporulation.

### Biofilm formation is modulated by *rsbW*.

**FIG 3:**
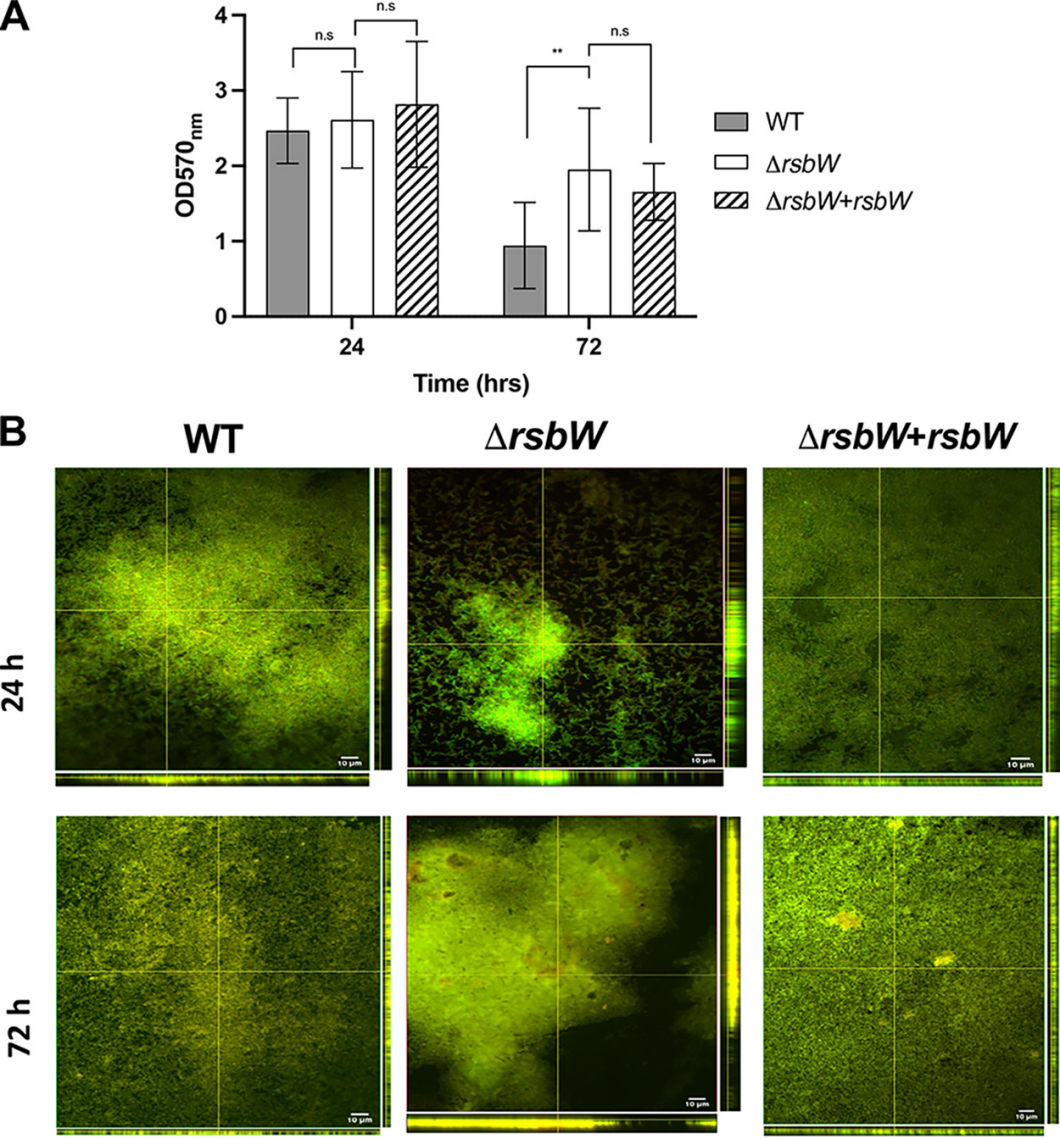
Biofilm formation is modulated by RsbW. (A) C. difficile strains were cultured in brain-heart infusion with 0.5% (wt/vol) yeast extract and 0.1% (wt/vol) l-cysteine (BHI-S) + 0.1 M glucose for 24 and 72 h in 24-well tissue culture-treated polystyrene plates, and biofilm biomass was quantified by crystal violet at OD_600nm_ (*N* = 3, with 3 technical replicates/experiment). (B) 24- and 72-h biofilms grown in chamber slides were stained with FilmTracer LIVE/DEAD. Orthogonal views of the biofilm z-stack depict biofilm thickness (*N* = 3, with 5 representative images/experiment). Error bars indicate SD. n.s., no significance; **, *P* < 0.01, as determined Student’s *t* test or Mann-Whitney U test.

C. difficile is known to produce a biofilm, which is a dense extracellular matrix that forms a physical barrier to dampen environmental stresses (34, 35). Biofilms formed by WT and Δ*rsbW* were quantified using crystal violet staining at 24 and 72 h. At 24 h (Fig. 3A), no significant difference was observed between the strains. However, at 72 h, Δ*rsbW* appeared to produce ~2-fold more biomass overall compared to the WT (*P* = 0.008).

To probe these differences further, biofilms were stained with a LIVE/DEAD dye and examined by confocal microscopy. Biofilms formed by the WT and Δ*rsbW* strain exhibited different characteristics (Fig. 3B) with the WT forming consistent, with thin biofilms at both 24 and 72 h and Δ*rsbW* forming more varied, thicker, but sparser biofilms. The complemented strain behaved in a similar manner to the WT strain; however, some thick biofilm structures were intermittently spotted (Fig. S7) and showed similar distribution to Fig. 3A. Thus, RsbW may play a role in modulating C. difficile biofilm formation.

### Δ*rsbW* adheres to epithelial cells better than the WT in a human *in vitro* gut model.

**FIG 4:**
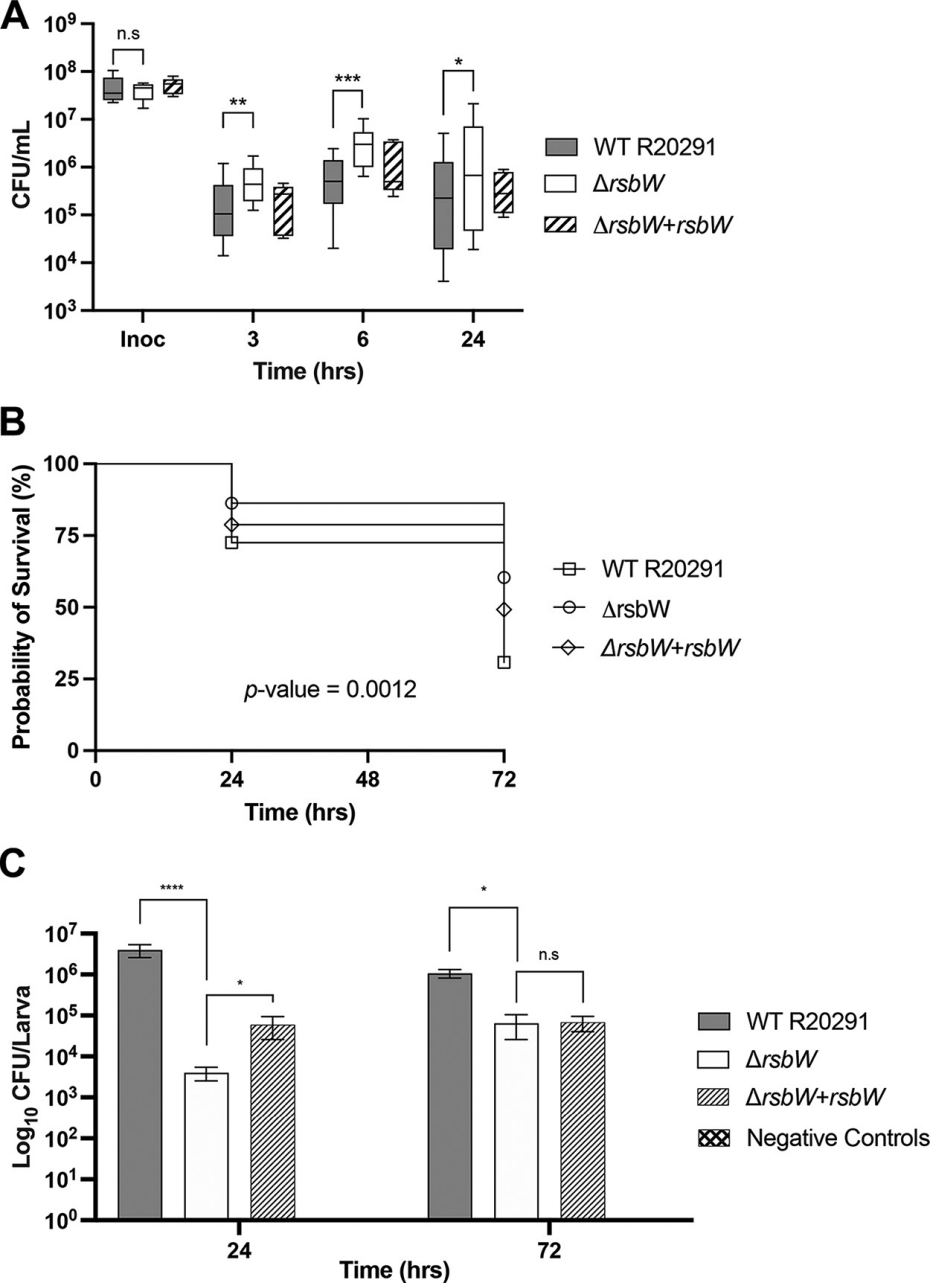
Δ*rsbW* demonstrates increased cell adhesion but decreased virulence. (A) WT C. difficile, Δ*rsbW*, and Δ*rsbW*+*rsbW* adherence to epithelial cells across 3, 6, 12, and 24 h after infection in an *in vitro* vertical diffusion chamber (VDC) infection model (*N* = 3, with 3 technical replicates/experiment). (B) Survival curve of Galleria mellonella infected with C. difficile WT, Δ*rsbW*, and Δ*rsbW*+*rsbW* strains across 24 and 72 h (N = 5, with 8 larvae/strain/time point). (C) Adherent bacterial population recovered from infected G. mellonella gut enumerated with CFU/mL on brain-heart infusion with 0.5% (wt/vol) yeast extract and 0.1% (wt/vol) l-cysteine (BHI-S) agar. Error bars indicate SD. n.s., no significance; *, *P* < 0.05; **, *P* < 0.01; ***, *P* < 0.001; ****, *P* < 0.0001, as determined by Mann-Whitney U test and log rank Mantel Cox test for the survival curve.

The gut mucosa subjects invading bacteria with a range of environmental insults. σ^B^ has been associated with increased colonization in a dixenic mouse infection model (17); however, its interactions with human epithelial cells have not been studied. Using an *in vitro* gut model previously used to probe for C. difficile bacterial adhesion (36), we compared the attachment of WT and Δ*rsbW* to a multicellular layer of Caco-2, HT-29 MTX E12, and CCD-18co cells in a vertical diffusion chamber (VDC). This dual compartment system allows bacterial growth in anaerobic conditions and keeps the epithelial cells in an oxygenated environment. VDCs were infected with WT or Δ*rsbW* at an multiplicity of infection (MOI) of 100:1 for 3, 6, and 24 h. Nonassociated bacteria were washed off, and adherent bacteria were enumerated by measuring CFU. Across each time point, more Δ*rsbW* was recovered from the monolayer compared to the WT (Fig. 4A). At 3 h, Δ*rsbW* was able to adhere ~2-fold more than the WT, increasing to an average 4-fold difference at 6 h. Although more variation was observed at 24 h, a similar average difference of 3.5-fold was observed between the WT and mutant strains.

### RsbW affects survival in a Galleria infection model.

An insect infection model was used to study the role of RsbW in virulence. Galleria mellonella, previously used to assess the larval outcomes with respect to phage therapy in the treatment of C. difficile infection (37, 38), is noted for ease of use and cost-efficiency. We infected ethanol-sterilized larvae with approximately 1 ×10^5^ CFU of WT, Δ*rsbW*, or *rsbW* complemented strain through oral gavage, and the infection outcome was monitored over 72 h. Any larvae that displayed immobility or melanization (brown and hardened blemishes) or had turned black were deemed deceased (38). Fig. 4B shows that G. mellonella infected with Δ*rsbW* displayed a higher survival rate at both 24 and 72 h (*P* = 0.0012) compared to those infected with the WT strain. At 24 h, larvae infected with WT R20291 had a survival rate of 73%, while Δ*rsbW* had 86%, but at 72 h, 70% of the larvae had succumbed to infection with the WT strain, as opposed to 40% when infected with Δ*rsbW*. The complemented strain showed an intermediate survival rate that was between the WT and the mutant strains. At each time point, the gut from each larva was extracted, and the CFU were enumerated (Fig. 4C). The bacterial numbers show a similar trend to the survival assay (Fig. 4B): at 24 h a 3-fold difference was observed between both strains. At 72 h, the difference between recovered bacterial loads decreased by 1-fold. While we report a regulatory role for RsbW in adherence, we see a decrease in recovered bacterial numbers, suggesting a lower rate of attachment to or survival within insect guts. This may indicate that other host factors may influence survival of a strain lacking RsbW in a complex gut environment.

### Expression of σ^B^-associated genes in Δ*rsbW*.

**FIG 5:**
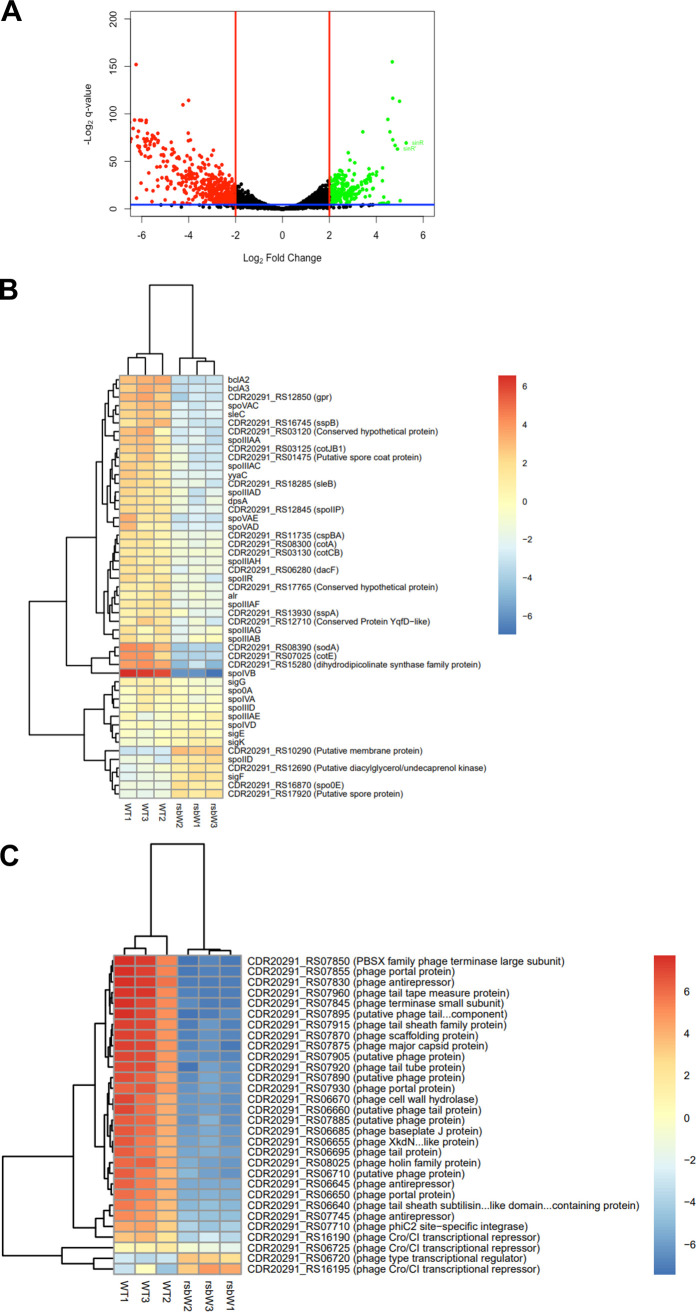
Bacterial transcriptomics of Δ*rsbW* reveals extensive differential expression compared with WT. (A) Volcano plot reveals differential gene expression when early stationary phase (10 h) Δ*rsbW* is compared to the WT (*N* = 3; *P* < 0.05). (B, C) Heat map representation of sporulation genes (B) and phage-associated genes (C) that were differentially expressed in Δ*rsbW*. Blue and red gradients indicate down- and upregulation, respectively, compared to WT.

As some of the phenotypes observed for the *rsbW* mutant were distinct from those reported for the σ^B^ mutant, we wanted to understand the effect of *rsbW* deletion on the σ^B^-modulated genes. The transcriptomic profiles of WT R20291 and Δ*rsbW* strains cultured in BHI-S to early stationary phase (10 h) were compared by RNA-Seq. Biological replicates of WT andΔ*rsbW* were each seen to cluster together in principal-component analysis (PCA) plots (Fig. S8A). Correlation analysis also showed low variation between samples (Fig. S8B). In total, 234 and 483 genes were up- and downregulated, respectively (Fig. 5A) in the Δ*rsbW* compared to the WT strain. The complete list of differentially regulated genes is included in Table S4. Surprisingly, majority of σ^B^-controlled genes previously reported in a C. difficile 630 σ^B^ mutant (where experiments were done under similar growth conditions) (17, 23), including genes responsible for oxygen tolerance, oxidative, and nitrosative stress responses and acid tolerance (Table S5), were unchanged or downregulated contrary to an upregulation one would expect if σ^B^ was “overexpressed”.

Among the 71 genes associated directly with sporulation (32), 17 genes were significantly downregulated in the Δ*rsbW* mutant (Fig. 5B), with an emphasis toward spore coat proteins (*cotE*, *BclA2*, *BclA3*, CDR20291_RS03125, and CDR20291_RS01475) (39, 40) and mid- to late-stage sporulation genes (*spoIVB*, *spoVAE*, *spoIIIAA*, *spoVAD*, and *spoVAC*). This transcriptional change to prevent spore maturation and release can be reflected in the sporulation frequency observed in Fig. 4A. While the expression of the global regulator Spo0A associated with spore formation (34) was unchanged, other regulators, including CodY and SigF, were upregulated in the mutant, and an ~5-log_2_ fold increase in expression was observed for the *sinRR′* locus (CDR20291_RS11605 and CDR20291_RS11610), which encodes a pleiotropic regulator that modulates sporulation, motility, and toxin expression (41, 42).

Flagella and pilin genes were also downregulated in the Δ*rsbW* mutant; however, no differences were seen between strains in motility assays with 0.3% agar (Fig. S9A). Interestingly, *tcdA* and *tcdB* were downregulated by 2-log_2_ fold and 0.5-log_2_ fold, respectively, and since direct control of the expression of toxins by σ^B^ has not been described, this could suggest an indirect regulation involving an additional regulator. Toxins A and B measurements from the WT and Δ*rsbW* planktonic cultures indicated a decrease in toxin production, although the differences were not statistically significant (Fig. S9B).

Few genes known to be associated with biofilm formation were differentially expressed, including a c-di-GMP-independent VaFE repeat-containing surface-anchored protein (*CDR20291_RS18550*) that was downregulated 2.6-log_2_ fold (Table S4) (43). Other surface protein genes were downregulated: *CDR20291_RS14700*, which encodes a c-di-GMP controlled surface protein, and *pilA1* (43, 44). Several cell wall protein (Cwp) genes, *cwp22*, *cwp18*, *cwp10*, and *cwp7*, were upregulated in Δ*rsbW*, and a couple of others, *cwp27* and *cwp5*, were downregulated. While the functions of many of these Cwps are not known, Cwp22 has pleiotrophic functions, including a role in cell adhesion (45). There was no significant change in the expression of σ^B^. Due to the overlapping stop-start codon, small transcripts of *rsbW* were counted; however, manual inspection of reads confirmed a *rsbW* deletion in the mutant.

Finally, it was interesting to see differential expression of many phage-associated genes (*p*_adj_ < 0.05) in Δ*rsbW*. Most of the phage-associated genes were from incomplete and intact phages ΦMMP04 and ΦC2, most of which were downregulated. The genes belonging to ΦMMP04 (Fig. 5C) are either unchanged or severely downregulated, possibly controlled by CDR20291_RS06720, a CI repressor. A similar transcriptomic profile can be observed in ΦC2 (Fig. S8D); however, the role of the Cro-CI bistable switch is less clear.

Thus, the RNA-Seq studies in 20291 C. difficile indicate that the absence of RsbW is associated with distinct transcriptional profiles with some changes similar to ones reported previously in C. difficile σ^B^ mutant. Some of the transcriptional changes observed could be attributed in part to the induction of other transcriptional regulators like SinRR′.

### Intracellular concentrations of σ^B^ are lower in unstressed Δ*rsbW*.

**FIG 6:**
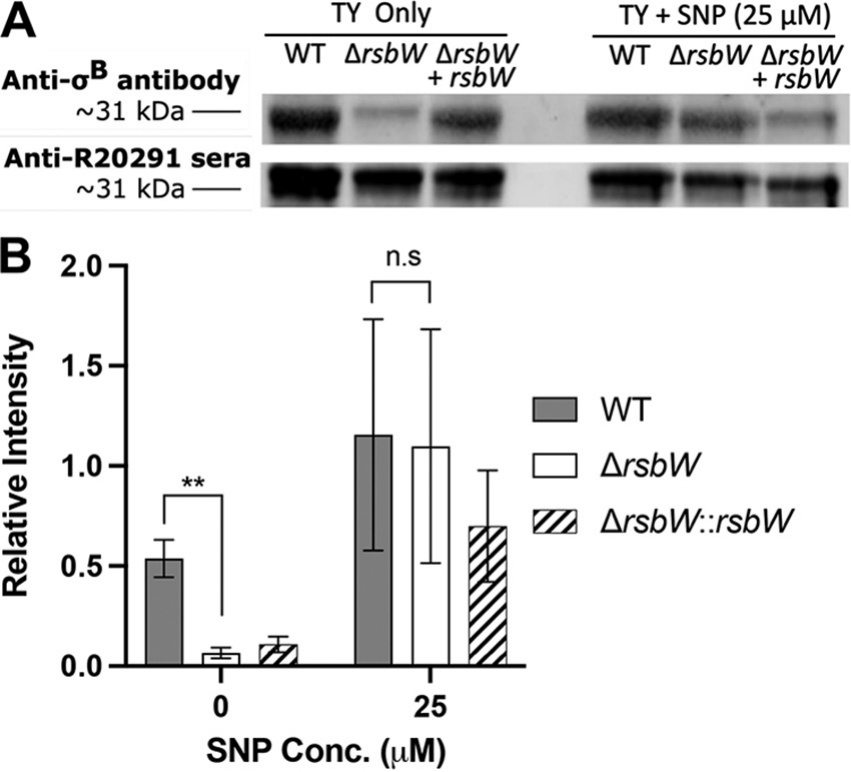
Immunoblotting shows altered relative concentrations of intracellular σ^B^ in Δ*rsbW.* (A) Bacterial whole-cell lysates from C. difficile strains C. difficile WT, Δ*rsbW*, and Δ*rsbW*+*rsbW* grown in tryptose yeast broth ± sodium nitroprusside (SNP) to early exponential phase (5 h) were analyzed by immunoblotting with anti σ^B^ or anti-R20291 sera (for normalization). The image is representative of three independent experiments. (B) Band intensities were calculated using Fiji (ImageJ) to determine relative intracellular σ^B^ concentrations at 5 h ±25 μM SNP. Error bars indicate SD. n.s., no significance; *, *P* < 0.05; **, *P* < 0.01, as determined by Student’s *t* test.

To understand phenotypes displayed by **Δ***rsbW* and its distinct transcriptomic profile, the intracellular concentrations of σ^B^ were compared between WT and Δ*rsbW* (anti-σ^B^ monoclonal antibody kindly provided by W.K. Smits, Leiden University Medical Centre). The relative quantities of σ^B^ were measured in planktonic cultures during exponential phase (5 h) in nonstressed and stressed conditions (induced by 25 μM SNP). Surprisingly, during nonstressed growth conditions, a 3-fold decrease (*P* = 0.0086) in σ^B^ was observed in Δ*rsbW* compared to the WT for exponentially growing bacteria (Fig. 6A and B). Conversely, when the bacteria were exposed to nitrosative stress, higher intracellular σ^B^ levels were seen for the WT, Δ*rsbW*, and complemented strains (Fig. 6A and B), but with no significant differences between strains. Variation was observed in phenotypic complementation, as artificial overexpression of σ^B^ was possibly counteracted by an undefined post-translational/regulatory/degradation mechanism. Furthermore, the SDS-PAGE protein profiles of total bacterial lysates revealed an altered abundance of proteins for Δ*rsbW* compared to the WT (Fig. S10A). Mass spectrometry analysis of bands that were prominently seen in the *rsbW* mutant showed a higher relative amount of two reverse rubrerythrins (CDR20291_RS07230 and CDR20291_RS07495) in Δ*rsbW* (Fig. S10B and C), which are known to be controlled by a σ^B^ promoter (29).

Thus, our data indicate that in the absence of stress, in cells lacking RsbW, σ^B^ is likely removed or degraded by an unknown σ^B^ degradation mechanism, which may explain the lack of growth defects in the Δ*rsbW* mutant, the transcriptional profiles, and some of the phenotypes observed. Under conditions of nitrosative stress, σ^B^ is not removed or degraded, indicating the necessity of σ^B^ in RNS detoxification.

## DISCUSSION

This study presents a distinctive insight into σ^B^ activation and control through the anti-sigma factor RsbW in C. difficile, based on findings from a deletion mutant that lacks this regulator. We report here that RsbW modulates many σ^B^-mediated stress responses, as evident from many stress-associated and survival phenotypes. However, the transcriptional profiles observed in the absence of RsbW were distinct yet similar to a σ^B^ mutant. This may be the result of lower intracellular σ^B^ levels in the *rsbW* mutant and may suggest stress-dependent post-translational control of σ^B^ levels in C. difficile.

To our knowledge, this is the first description of a targeted *rsbW* bacterial mutant. Previous studies have attempted to create isogenic *rsbW* mutants in other bacteria like Bacillus, but in-depth studies have not been successful due to a toxic phenotype (20, 25). In Staphylococcus aureus, where RsbW and σ^B^ are expressed through coupled translation, the *rsbW* mutant showed a σ^B^ mutant phenotype (46). Unlike other *Bacillota*, the σ^B^ operon in C. difficile is under basic control by the housekeeping σ^A^, without a σ^B^ promoter and hence devoid of a positive feedback loop. Thus, we would expect the absence of RsbW in C. difficile would lead to unbound σ^B^, in an “always on” manner, without a fitness defect.

While as expected the Δ*rsbW* did not have a fitness defect in normal growth conditions, it was able to respond quicker to low oxygen concentrations, detoxify ROS/RNS stress and acidic environments, and thus survive better under these conditions. This can be observed in growth curves in 1% oxygen, 25 μM SNP, and pH 5. A C. difficile 630 sigB mutant was previously reported to be more sensitive to oxidative and nitrosative stresses; hence, an *rsbW* mutant with sigB readily available would be expected to be more resistant to such stresses. However, more extreme conditions were required to elicit a difference between the WT and the Δ*rsbW* mutant, as the WT strain was able to tolerate higher concentrations than described in the literature (17). The differences seen between ROS inducers H_2_O_2_ and paraquat (methyl-viologen) may be due to the requirement by the latter of an additional enzyme to neutralize it. In the detoxification of paraquat, B. subtilis employs superoxide dismutase (*sodA*), which catalyzes the disproportionation of O_2_^−^ into O_2_ and H_2_O_2_ (47). The WT and complemented strains likely succumb to the effects of superoxide before the stress response genes are activated. However, *sodA* in C. difficile has been associated only with spore coat formation or maturation (39) and not with aerobic stress management (48, 49). Instead σ^B^-associated desulfoferrodoxin, reverse rubrerythrins, and NADH-rubredoxin reductases have been described as remedial agents (17). In line with this, some reverse rubrerythrins were constitutively produced at high levels in Δ*rsbW*, as shown by mass spectrometry. The availability of the reverse rubrerythrins allows the mutant to tolerate low oxygen and oxidative stress better, enabling quicker growth.

Sporulation and biofilm formation, both phenomena that help the bacterium persist under conditions of stress, were affected by the absence of RsbW. Sporulation was clearly decreased in the absence of RsbW, and this was supported by the transcriptional analyses that showed downregulation of late-stage sporulation genes that are involved in the maturation and release of spores. This aligns well with the increased sporulation rate reported for C. difficile σ^B^ mutants (17, 50). An increased biofilm formation was observed in the absence of RsbW, and the differences in biofilm formation were maximal at later stages of biofilm growth (72 h), indicating a potential role for nutritional stress, a build-up of metabolic waste products, and medium acidification in this phenotype. A defect in the ability to form biofilms was not seen with C. difficile σ^B^ mutants (17), although σ^B^ has been associated with biofilm formation in S. aureus, which controls fibronectin-binding protein A expression (51, 52). C. difficile R20291 encodes orthologs of fibronectin-binding proteins, such as FbpA, which is involved in early biofilm formation and colonization (43, 53). However, *fbpA* expression was unchanged in Δ*rsbW*. An increased expression of some Cwp genes, such as *cwp22* (associated with adhesion and biofilm formation) and several uncharacterized Cwps that potentially affect adhesion, could contribute to the increased biofilm formation by Δ*rsbW*.

Bacteria are exposed to different types of stresses, including soluble mediators and antimicrobial peptides, when they are in contact with mammalian cells. We see an increased adhesion with Δ*rsbW* in an *in vitro* gut model in which in addition to cell-associated factors, bacteria are likely also exposed to low levels of oxygen and metabolic stress. A 10-fold decrease in colonization was previously reported when σ^B^ was inactivated in 630Δerm; hence, we would expect that the Δ*rsbW* under stress is able to associate more quickly with the epithelium and to multiply earlier. Although it displayed an increased adhesive ability in an *in vitro* human gut model, interestingly Δ*rsbW* did not infect as well as the WT in a G. mellonella infection model across 24 and 72 h. The insect gut could be a more complex environment, needing a better regulation of stress responses, which was affected in the absence of RsbW. On the other hand, as noted previously, a difference between the WT and Δ*rsbW* was observed only in extreme stress conditions, which the insect infection model might not be able to provide. Therefore, the lower recovered bacterial numbers could be the result of the partially stressed bacterium possessing a low intracellular concentration of σ^B^.

The transcriptomic profile of Δ*rsbW* unexpectedly revealed the lack of differential expression of several genes in the σ^B^ regulon as previously described (17, 23). Whole-genome sequencing of the WT and Δ*rsbW* strains indicated no secondary mutations in the Δ*rsbW* (data not shown). Although as expected the transcription of the *sigB* gene did not change *per se*, the lower intracellular concentration of σ^B^ in the Δ*rsbW* in a nonstressed state and the RNA-Seq profiles suggest that the Δ*rsbW* in part mimics a σ^B^ deletion mutant. In this study, transcriptomic profiles of R20291 Δ*rsbW* were compared to a σ^B^ regulon from a 630 strain. It is possible that there is strain-specific σ^B^ regulation, although previous studies comparing σ^B^ mutants in R20291 and 630 backgrounds did not see major phenotypic differences (17, 18, 23). Moreover, given the lower σ^B^ levels in the nonstressed Δ*rsbW*, a direct comparison with a R20291 mutant likely will not help clarify the RsbW-specific profiles. We also cannot exclude that RsbW may interact with sigma factors other than σ^B^ and mediate control of additional sets of genes.

One of the regulators with the highest levels of upregulation in the Δ*rsbW* was the SinRR′. SinR and SinR′ are transcribed as a single transcript but have not been directly associated with σ^B^ control, nor has a σ^B^ promoter been identified upstream of the locus (17). The *sinRR*′ expression can be suppressed by Spo0A, as a phosphorylated Spo0A (Spo0A~P) can bind to the only promoter sequence upstream of this locus (54). While this locus and σ^B^ may contribute to Δ*rsbW* phenotypic responses, SinR regulates the transcription of other pleiotropic regulators such as σ^D^, Spo0A, and CodY, which can regulate many other genes in C. difficile (41, 55–57). In B. subtilis, Spo0A is negatively controlled by Spo0E through dephosphorylation; furthermore, Spo0E is positively controlled by a σ^B^ promoter (50). Additionally, it has been suggested that SinR is able to regulate *spo0A* expression through an unknown mechanism (54). The SinR homolog in B. subtilis functions as a repressor for the spo0A promoter (58). Our transcriptomic data indicate no changes in *spo0A* expression, but *spo0E* is upregulated by ~1.5 log_2_ fold. Hence, we propose that σ^B^ regulates the *sinRR*′ locus through Spo0E; Spo0E dephosphorylates Spo0A~P to Spo0A, subsequently derepressing the *sinRR*′ locus.

Another intriguing finding was the downregulation of several phage structural and functional genes in the Δ*rsbW* mutant. Notably, a downregulation of predicted “lytic” genes was evident. Phaster annotation of R20291 genome describe two incomplete and one complete prophage regions, including the *Myoviridae* phages ΦMMP04 and ΦC2 (59, 60). However, their association with σ^B^ has not been reported. Integrases and Cro control the lytic lifestyle of prophages, while CI (represses *int*) maintains lysogenic stability (61, 62), with Cro/CI forming a double-negative feedback loop. The effect of σ^B^ on both proteins is the clearest in ΦMMP04, in which the gene encoding CI (CDR20291_RS06720) is upregulated and Cro (CDR20291_RS06725) is downregulated. Although ΦMMP04 does not have an annotated integrase in the prophage region (60), the majority of its predicted lytic genes are downregulated, presumably as a consequence. In ΦC2 (Fig. S8D), the Cro/CI repressors are not annotated; however, several helix-turn-helix transcriptional regulators, bearing homology to Cro/CI, were differentially expressed, and *int* (CDR20291_RS07710) and ~50 genes in the prophage region were downregulated (Table S4). Therefore, σ^B^ could potentially exert control for lysogenic stability, which was previously associated with σ^H^ in S. aureus (63).

It was surprising that there was a significantly lower concentration of intracellular σ^B^ in Δ*rsbW* in a nonstressed condition, as one would expect that σ^B^ accumulates in the absence of RsbW. However, similar concentrations to the WT were observed when exposed to stress conditions (induced by SNP). A previous study in B. subtilis reported a 10% decrease in σ^B^ under nonstressed conditions, although relative levels of σ^B^ were not measured under stress (19). A lower concentration of unbound σ^B^ would result in a lower or negligible expression of unnecessary genes and minimally affect bacterial fitness. This may suggest an unknown proteolytic/σ^B^ degradation system that is induced during nonstressed states. Caseinolytic proteases (Clps) are one of the main systems employed by bacteria for regulatory protein homeostasis (64), Clp homologs in *Bacillota* and C. difficile have been associated with growth and virulence (65–67). While it is possible that Clp could control σ^B^, we cannot exclude other post-transcriptional control mechanisms that control translation of σ^B^. It is also possible that the sequestration of σ^B^ by RsbW could prevent σ^B^ degradation; unbound σ^B^ could lack the steric shielding that RsbW provides.

Similar to *Bacillus* spp., in C. difficile, σ^B^ is known to play a key role in various stress responses (10, 68). Although RsbW binds to σ^B^ in both species controlling σ^B^ activity post-translationally, there are clear differences in how σ^B^ gene expression is regulated in both species (17, 69, 70). Here, we report that C. difficile Δ*rsbW* constitutively expresses σ^B^ at low levels in the absence of stress and displays unique phenotypes in stress response, persistence, and infection. Our study reveals that σ^B^ levels are carefully controlled in C. difficile and may involve post-translational mechanisms other than the partner-switching mechanism in both nonstress and stress conditions. Further studies into factors controlling intracellular σ^B^ levels could provide insight into C. difficile survival under conditions of stress.

## MATERIALS AND METHODS

### Bacterial strains and culture conditions.

The bacterial strains and plasmids used in this study are listed in the Table S1. C. difficile strains were grown in prereduced brain-heart infusion (Oxoid, UK) supplemented with 0.5% (wt/vol) yeast extract and 0.1% (wt/vol) l-cysteine (Sigma Aldrich, UK) (BHI-S), tryptone yeast (TY) medium, or in DMEM-10. Media were also supplemented with antimicrobials; cefoxitin (8 μg/mL), d-cycloserine (250 μg/mL), and thiamphenicol (15 μg/mL) where required. Complemented strains were induced with anhydrotetracycline (20 ng/mL) for the *Ptet* promoter containing pRPF185 derivatives in this study (71). Cultures were grown under anaerobic conditions (80% N_2_, 10% CO_2_, and 10% H_2_) in a Don Whitely Scientific MG500 Anaerobic Workstation (Yorkshire, UK). Escherichia coli strains were grown in LB medium supplemented with chloramphenicol (15 μg/mL) where necessary.

### Construction of Δ*rsbW*.

The allele exchange system from Cartman et al. (72) was employed in the generation of the Δ*rsbW* mutant in C. difficile strain R20291. The specific details of the generation of mutant and complement strains can be found in the supplemental materials. The allelic exchange cassette was generated “in frame,” flanking the gene of interest and cloned into the pMTL-SC7315 vector. The construct was transformed into E. coli DH5α by heat shock and then electrocompetent E. coli donor strain CA434 and subsequently conjugated into R20291. Transconjugants, first and second crossover mutants, were selected and Sanger sequenced to confirm successful generation of mutants.

### Fitness and overexpression assays.

Stationary-phase cultures of bacteria were diluted to an OD_600nm_ of 0.05 in a large volume prereduced BHI-S, TY broth, or DMEM-10 and grown in anaerobic conditions, and OD_600nm_ was measured on a Novaspec Pro spectrophotometer (Biochrom, USA). Overexpression of RsbW was induced with 200, 1,000, and 2,000 ng/mL of anhydrotetracycline (aTc) in BHI-S broth supplemented with thiamphenicol. The viability of bacteria and plasmid stability was also measured via a spot assay on BHI-S plates supplemented with/without thiamphenicol and/or aTc; stationary-phase cultures were standardized to an OD_600nm_ of 1.0 and serially diluted in prereduced sterile phosphate-buffered saline (PBS), and 10 μL of each dilution was dispensed onto agar plates (150 × 150 × 15 mm, Sarstedt, Germany). CFU were counted after incubation for 24 h in anaerobic conditions.

### Oxygen tolerance assay.

To measure tolerance and growth in an aerobic environment, the strains were grown in soft agar tubes as previously described in literature (17, 73). A total of 20 μL of stationary-phase cultures (grown anaerobically in TY medium) was mixed into tubes containing 0.4% TY agar. Strains were grown aerobically at 37°C for 24 h, and the growth inhibition was measured by two independent lab members.

Strains were assessed for their growth at 1% oxygen in a Don Whitley Scientific MA500 VAIN Workstation, stationary-phase cultures were diluted to an OD_600nm_ of 0.1 in prereduced TY medium, and growth was measured every 2 h for 12 h. In parallel, stationary-phase cultures were diluted to an OD_600nm_ of 1.0, serially diluted, spotted on prereduced TY agar, and incubated for 24 h.

### Acidic stress assay.

Stationary-phase cultures were diluted to an OD_600nm_ of 0.1 in prereduced BHI-S adjusted to pH 4, 5, 6, and 7. Bacterial growth was measured every 2 h in a spectrophotometer. Viable cells were enumerated onto prereduced BHI-S agar plates at 0, 6, 12, and 24 h.

### Oxidative and nitrosative stress detoxification assay.

To assess oxidative stress, stationary-phase cultures were standardized to a McFarland Standard of 1.0 and plated onto prereduced BHI agar plates. Sterile 10-mm blank antibiotic disks (Oxoid, UK) were place on the plate, and 10 μL of (1, 2, 4, and 9.8 M) H_2_O_2_ and (2, 4, and 6 M) methyl viologen (Sigma-Aldrich, UK) was dispensed onto each separate disk. Inhibition of bacterial growth was measured with the zone of inhibition (mm) after 24 and 48 h of incubation.

A spotted dilution assay was conducted in a similar manner to that of Kint et al. (17). Stationary-phase cultures were diluted in prereduced sterile PBS, and 10 μL were spotted onto prereduced TY media supplemented with 200, 500, 1,000, and 1,500 μM sodium nitroprusside (SNP). After 24 h of incubation, bacterial growth was enumerated via CFU/mL and compared to the control agar plates (no SNP).

### Motility assay.

Motility assays were conducted as described in literature (74). Briefly, 3 μL of stationary-phase culture was spiked into the center of 0.3% BHI-S agar and incubated unturned at 37°C. At 24 and 48 h, the diameter of each sample was measured for motility.

### Sporulation and germination assays.

Sporulation assays were conducted as described by Edwards and McBride (75). Spores were also enumerated by phase-contrast microscopy on a DM18 (Leica Microsystem, Germany). 2 μL of each sample (controls and post-treatment) were dispensed onto 1% agar pads. Five randomly selected representation images were captured, and the percentage of spores to total cells was calculated.

### Biofilm formation.

The quantification of biofilm biomass by crystal violet (CV) was done in a similar manner to the protocol described by Dapa et al. (34). Stationary-phase cultures were standardized to an OD_600nm_ of 0.1 and subcultured until OD_600nm_ of 0.5 in BHI-S + 0.1 M glucose (BHI-SG). The cultures were subsequently diluted to an OD_600nm_ of 0.05, and 1 mL was dispensed into prereduced 24-well tissue culture-treated plates (Falcon, USA). The plates were incubated for 24 or 72 h (parafilm was used to secure the lid and plate to avoid excessive evaporation). At each time point, each well was gently washed twice with prereduced sterile PBS, allowed dry for 30 min, and stained with 1 mL 0.2% filter-sterilized CV for 30 min. Excess CV was removed, the wells were washed twice with 1 mL PBS and destained with 1 mL of methanol for 30 min. The destained CV was diluted 1:1, 1:10, and 1:100 in methanol, and OD_570_ was measured immediately.

For microscopy, biofilms were grown in the Nunc LabTek II Chamber Slide system (ThermoFisher) with 1 mL of diluted culture. After 24 and 72 h, planktonic medium was carefully removed and washed twice with 0.1% saponin (wt/vol) and incubated with FilmTracer LIVE/DEAD biofilm viability kit (Invitrogen, USA) following the manufacturer’s instructions. The dyes were washed twice with prereduced PBS, and the biofilm was fixed with 4% paraformaldehyde (wt/vol) for 15 min. Samples were washed twice with 500 μL sterile water and imaged using with a Perkin Elmer dual-camera spinning disk confocal microscope. Z-stacks of each biofilm were taken at the recommended excitation/emission spectra with increments of 0.5 μm. Five representative images were taken and analyzed using Fiji (ImageJ).

### Toxin assay.

Stationary-phase cultures in BHI-S were subcultured into fresh prereduced BHI-S and grown until the early stationary phase (10 h). Each sample was normalized by OD_600nm,_ and the toxin was quantified by separate detection of C. difficile toxin A and B enzyme-linked immunosorbent assay (ELISA) (tgcBIOMICS, Germany) following the manufacturer’s instructions.

### RNA extraction.

Stationary-phase cultures were mixed with RNA protect (Qiagen, UK) at a ratio of 1:2 and pelleted at 5,000 × *g* for 10 min. The samples were resuspended with 600 μL ice-cold LETS buffer (0.1 M LiCl, 0.01 M Na_2_EDTA, 0.01 M Tris-Cl [pH 7.4], and 0.2% SDS) into lysing matrix B tubes (MP Biomedicals, China) and run on a FastPrep-24 5g (MP Biomedical, China) for a total of 6 cycles: 30 s at 6.5 m/s and 180 s on ice. The tubes were subsequently centrifuged at the highest speed at 4°C for 10 min, and the supernatant was transferred to nuclease-free tubes. TRIzol-chloroform RNA extraction was performed, 1 mL of TRIzol reagent (ThermoFisher Scientific, UK) was added per 1 ×10^7^ CFU/mL bacteria and incubated at room temperature (RT) for 5 min. 500 μL of chloroform was added, further incubated at RT for 10 min, and centrifuged at 16,000 × *g* at 4°C for 15 min. The supernatant was dispensed into a new nuclease-free tube, and 500 μL of isopropanol was added and incubated at RT for 10 min. Samples were centrifuged at 12,000 × *g* at 4°C for 10 min, and the pellet was washed with fresh 70% ethanol and resuspended with nuclease-free water. TURBO DNase (Invitrogen, UK) was used to removed contaminating genomic DNA according to the manufacturer’s protocol. The treated samples were cleaned with 2.5 mM nuclease-free LiCL (Invitrogen, UK) according to the manufacturer’s protocol. The purity of each sample was analyzed with the RNA Pico 6000 assay protocol on an Agilent 2000 bioanalyzer, according to the manufacturer’s protocol (Agilent, USA).

### RNA sequencing and analysis.

RNA samples were sent to Novogene Ltd. for rRNA removal and cDNA sequencing on the Illumina NovaSeq 6000 platform. Then 150-bp paired end sequences were analyzed with FastQC, with approximately 10 million reads/sample. The reads were mapped to C. difficile R20291 reference genome (NC_013316.1) with Bowtie2 (version 2.4.5) (76) and read counts generated using Samtools (1.13) and Bedtools (2.30.0) (77). DESeq2 was used to calculate differential gene expression using a negative bionomial distribution (78). The genes were deemed significant when the log_2_ fold change was ≥ −2 or ≤2 and the adjusted *P* value was ≤ 0.05. The results were visualized with R packages ggplot2 (https://cran.r-project.org/web/packages/ggplot2/index.html) and pheatmaps (https://cran.r-project.org/web/packages/pheatmap/index.html). All sequencing reads were deposited to the European Bioinformatics Institute (accession number E-MTAB-12114).

### *In vitro* gut model infections.

To assess bacterial adhesion to epithelial cells, the experiment was carried as previously described (36). Stationary-phase cultures of bacterial strains grown in BHI-S, standardized to an OD_600nm_ of 1.0 in DMEM-10, and incubated at 37°C in anaerobic conditions for 1 h. The inoculum was enumerated via CFU/mL. The Snapwell inserts, containing polarized Caco-2, HT29, and CCD 18co myofibroblasts, were each sandwiched between two compartments of the vertical diffusion chamber (VDC) (Harvard Apparatus, UK). 3.5 mL of medium of prewarmed DMEM-10 was added to the basolateral compartment, while the apical compartment was infected with the diluted, acclimatized bacterial strains at an MOI of 100:1. Aerobic and anaerobic gas was pumped into each respective compartment at approximately 25 lb/in^2^, with 15 to 20 cc/mm/half-cell. The bacterial culture on the apical compartment was washed with PBS and replaced with prereduced DMEM-10 at 3 h postinfection. At each desired time point, the Snapwells with adhered bacteria were washed twice with prereduced 1 mL PBS and lysed with prereduced 1 mL sterile water in anaerobic conditions. Adhesion was quantified through serial dilution of Snapwell lysates onto BHI-S agar.

### G. mellonella infection model for virulence.

G. mellonella (Livefoods, UK) were stored at 4°C and used within 1 week. The larvae were filtered by their skin blemishes and their ability to self-upright, weighed (0.25 to 0.30 g), and swab-sterilized with 70% ethanol. Stationary-phase cultures were subcultured in prereduced BHI-S and allowed to grow to an OD_600nm_ of ~0.3. The cultures were centrifuged at 2,400 × *g* at 4°C for 5 min and resuspended in ice-cold BHI-S to limit bacterial growth. A total of 10 μL was administered to each treatment group (of eight larvae) via oral inoculation between the mandibles using long gel loading tips (Eppendorf, UK). At each desired time point, the survival rate was numerated through visual observation based on melanization and larval movement, with black/brown, immobile insects constituting death (79). Larvae were placed on ice (to limit movement) and dissected dorso-ventrally with scissors to obtain gut contents. The extracted guts were placed in 100 μL ice-cold PBS, vortexed at 3,000 rpm for 1 min, and enumerated on BHI-S agar.

### Immunoblotting.

C. difficile strains were subcultured in BHI-S, TY, and TY supplemented with 25 μM SNP for 5 h, in a similar manner as described previously. A total of 10 mL of each sample was placed on ice and centrifuged at 5,000 × *g* for 5 min at 4°C. The supernatants were discarded, and the pellets were washed with ice-cold PBS. Bacterial cells were lysed with a freeze-thaw cycle (71), and the pellets were subsequently resuspended in ice-cold PBS with 10 μL/mL Halt protease inhibitor cocktail (ThermoFisher, USA) according to the manufacturer’s instructions. After incubation at 37°C for 1 h, the pellet was washed and resuspended in sterile water, and the protein concentration was quantified by Qubit (ThermoFisher, USA). The samples were normalized to a total concentration of 10 ng, loaded onto a 10% Mini-PROTEAN TGX stain-free precast gel (Bio-Rad, USA), and transferred onto polyvinylidene difluoride (PVDF) using standard methods. The membranes were blocked with 5% bovine serum albumin (Merck Millipore, USA) in TBST (1× Tris-buffered saline, 0.1% Tween 20 [vol/vol]), probed with affinity purified anti-σ^B^ (23) diluted 1:500 in bovine serum albumin (BSA) overnight, followed by anti-rabbit horseradish peroxidase (HRP)-linked secondary antibody (Cell Signaling Technology, USA). The blots were stripped using stripping buffer (0.2 M glycine, 0.1% SDS [wt/vol], 1% Tween 20 [vol/vol], and adjusted to pH 2.2), washed thrice, blocked overnight with 5% BSA, and probed with anti-R20291 sera.

Crude protein lysates stained with Coomassie brilliant blue R-250 (Bio-Rad, USA) were sent for mass spectroscopy at the Warwick Proteomics Research Technology Platform. The gels were digested (80) and processed with an Orbittrap Fusion with UltiMate 3000 RSLCnano system (Thermo Scientific).

### Statistical analysis.

All data were subjected to the Anderson-Darling or Shapiro-Wilk normality test (dependent on the sample size). To assess significance between two treatment groups, two-tailed Student’s *t* test and Mann-Whitney-Wilcoxon test were used. The significance of percentage survival was examined with the Kaplan-Meier estimator. Significance was denoted as follows: *, *P* ≤ 0.05; **, *P* ≤ 0.01; ***, *P *≤ 0.001; ****, *P* ≤ 0.0001; and n.s., no significance.
