# Supplementary material for: Regulatory Role of Anti-Sigma Factor RsbW in Clostridioides difficile Stress Response, Persistence, and Infection
Source: J Bacteriol. 2023 Apr 26;205(5):e00466-22. doi: 10.1128/jb.00466-22 (PMC10210984; doi:10.1128/jb.00466-22)
Supplement: Supplemental file 2 — Supplemental text, Tables S1 to S3 and S5, and Fig. S1 to S10. Download jb.00466-22-s0002.pdf, PDF file, 8.3 MB [file jb.00466-22-s0002.pdf]

Supplementary Material

Generation of Δ*rsbW*

C. difficile R20291Δ*rsbW* were generated using allelic exchange system (Cartman et al., 2012). Two homologous regions flanking either side of the *rsbW* gene (approximately 500 bp) were created in R20291 and the genomic DNA was isolated with DNeasy Blood and Tissue kit (Qiagen, Germany). These homologous regions were amplified using primer couples F1_*rsbW*/R1_*rsbW* (upstream region) and F2_*rsbW*/F2_*rsbW* (downstream region) with Phusion High-Fidelity DNA polymerase (NEB, USA) and restriction sites *PmeI* and *EcoRI* were affixed at the 5' and 3' ends respectively. One step ligation with Rapid DNA Ligation KIT (Roche, USA) was used to clone the two flanking regions into the PmeI site of pMTL-SC7315, to generate pMTL-SC7315-*rsbW*. This would generate an in-frame deletion of the open reading frame of *rsbW* (nucleotides 10-401). pMTL-SC7315-*rsbW* was transformed into *E. coli* DH5α (Invitrogen, USA). Transformants were selected on LB agar supplemented with 25 µg/mL chloramphenicol, subcultured in LB broth supplemented with 12.5 µg/mL chloramphenicol. pMTL-SC7315-*rsbW* was purified and screened with PmeI restriction digestion (NEB, USA) and amplified with primer pair SC7-F1/SC7-R1. DNA sequence was confirmed with Sanger sequencing using SC7-F/SC7-R.

pMTL-SC7315-*rsbW* was conjugated into *E. coli* CA434 using electroporation and selected on LB agar supplemented with 25 µg/mL chloramphenicol. R20291 and CA434/pMTL-SC7315-*rsbW* were grown overnight in BHI-S and LB broth with 25 µg/mL chloramphenicol where appropriate. 1 mL of stationary culture of CA434/pMTL-SC7315-*rsbW* was pelleted and washed with 500 µL sterile PBS. The conjugal pellet was resuspended with 200 µL of R20291 stationary culture, plated onto pre-reduced BHI-S agar plates and incubated in anaerobic conditions for 24 h at 37 °C. Colonies were collected with 500 µL PBS and plated onto BHI-S supplemented with 250 µg/mL D-cycloserine, 8 µg/mL cefoxitin and 15 µg/mL thiamphenicol. Plates were incubated in anaerobic conditions for 24-72 hrs at 37 °C, and transconjugants were restreaked onto similar plates. Single crossover events were isolated, confirmed by PCR

(RsbW_contr_F/SC7_R and RsbW_cont_R/SC7_R) and restreaked onto non-selective BHI-S agar for 96 hrs. Colonies were harvested in 500 µL sterile PBS, serially diluted and 100 µL spotted onto *C. difficile* defined minimal medium supplemented with 50 µg/mL fluorocytosine. Second crossover mutants were isolated and patch plated onto BHI-S supplemented with D-cycloserine, cefoxitin and thiamphenicol. Fluorocytosine-resistant and thiamphenicol-sensitive clones were confirmed with PCR (F1_*rsbW*/R2_*rsbW*) and Sanger sequencing.

To generate the complemented strain, the *rsbW* gene was amplified from *C. difficile* genomic DNA cloned into *SacI and BamHI* sites of a tetracycline-inducible plasmid, pRPF185 (kind gift from Robert Fagan), replacing the *gusA* gene. RsbW gene sequence was confirmed by sanger sequencing.

Supplementary Figures

Figure S1. Schematic diagram of the σ^B operon and the activation of σ^B.

A) Genetic organisation of the σ^B operon and *rsbZ* in *C. difficile* R20291. The σ^B operon and *rsbZ* locus are controlled by σ^A. B) Activation of σ^B-dependent genes. RsbZ is believed to sense stress and dephosphorylate RsbV. RsbW preferentially binds to RsbV, which releases σ^B from the control of RsbW and subsequently directs RNA polymerases to σ^B promoter sites.

Figure S2 Δ*rsbW* does not display any fitness defect in a non-stressed state.

Growth curves of *C. difficile* strains *C. difficile* WT, Δ*rsbW* and Δ*rsbW*+*rsbW* grown for 12 h in A) BHI-S, B) TY and C) DMEM media. D) Growth profiles were analysed with the GrowthCurver R package to calculate the generation time. N=3, error bars indicate SD.

Figure S3. Overexpression of *rsbW* does not induce cellular toxicity.

A) Overnight cultures of *C. difficile* WT, Δ*rsbW* and Δ*rsbW*+*rsbW* were normalised to OD_{600} 0.1 and spot diluted onto BHI-S agar with/without thiamphenicol/anhydrotetracycline. Viable bacteria were enumerated to quantify B) bacterial fitness and toxicity of anhydrotetracycline and C) plasmid-loss mediated survival. N=3 (3 technical replicates/experiment). Error bars indicate SD, no significance was denoted with n.s. and significance with *p-value < 0.05 as determined by Mann-Whitney U test

Figure S4. Δ*rsbW* does not confer increased tolerance to oxidative tolerance in agar.

Overnight cultures of *C. difficile* WT, Δ*rsbW* and Δ*rsbW*+*rsbW* were spiked onto 0.4% TY agar and grown aerobically for 24 h. A) Image of the zone of inhibition between the top of bacterial growth and the agar. B) The zone of inhibition was measured for each tube and the distribution was graphically represented. Data analysis was conducted with a Student's t-test. No significance was denoted with n.s.

Figure S5. No alteration in growth is observed when *C. difficile* strains subjected to pH 4, 6 and 7.

A) Growth profile of *C. difficile* WT, Δ*rsbW* and Δ*rsbW*+*rsbW* grown in BHI-S media calibrated to pH 4, 6 and 7. B) At each timepoint, cultures were grown on BHI-S agar plates to enumerate viable colonies.

Figure S6. Bacterial stress tolerance to ROS (H_2O_2) and RNS (sodium nitroprusside).

A) *C. difficile* WT, Δ*rsbW* and Δ*rsbW*+*rsbW* were grown on bacterial lawns were exposed to 1, 2, 4 and 9.8 M of H_2O_2 on 10 mm disks. The diameter of the zone of inhibition was measured. B) Viable bacteria were enumerated from *C. difficile* strains grown on TY agar plates supplemented with 0, 0.2, 0.5 and 1 mM sodium nitroprusside. Differential survival was

calculated with fold change between the bacterial grown with and without SNP. N=3 (3 technical replicates/experiment). Error bars indicate SD, no significance was denoted with n.s and significance with * *p*-value < 0.05 as determined by Student's t-test and Mann-Whitney U test.

Figure S7. RsbW alters biofilm thickness at 72 h.

The voxel depth from the confocal microscopy images of each biofilm was calculated in Fiji (ImageJ). N=3 (3 technical replicates/experiment). No significance was denoted with n.s and significance with * = *p*-value < 0.05. as determined by Mann-Whitney U test.

Figure S8. Transcriptomic analysis of Δ*rsbW* vs WT

Biological replicates of the RNAseq data for *C. difficile* WT and Δ*rsbW* were analysed with A) Principal component analysis and correlation coefficient analysis of B) WT and C) Δ*rsbW*.

Figure S9. Δ*rsbW* does not significantly alter bacterial ability to swim and produce toxin.

A) Bacterial motility was measured on soft agar plates over 24 h and 48 h on 0.3% agar for swimming (3 technical replicates/experiment) B) Planktonic bacterial cultures were grown for 10 h, and toxin concentration were examined using ELISA for toxin A or B. N=3 (3 technical replicates/experiment). No significance was denoted with n.s. as determined by Mann-Whitney U test.

Figure S10. Mass spectrometry reveals constitutive expression of σ^B-controlled rubrerythrins in Δ*rsbW*.

A) Raw protein lysates obtained from *C. difficile* WT, Δ*rsbW* and Δ*rsbW*+*rsbW* that were grown to early exponential and stationary phase in BHI-S or TY medium. Samples were run through 12.5% polyacrylamide gel and stained with Coomassie blue. Excised bands were at ~21 kDa (denoted with white arrows) were digested and analysed by mass spectrometry, revealing B) a distribution of normalised total protein spectra and C) differential abundance of rubreythrins in both BHI-S and TY.

Table S1 Strains and plasmids used within this study

Table S2 Oligonucleotides used within this study

Table S3 Generation Time of *C. difficile* strains grown in pH 4-7

Table S4 Complete list of differentially regulated genes in rsbW compared to the WT 20291 strain.

Table S5 Comparison of changes in gene expression in σ^B controlled genes between a Δ*rsbW* 20291 strain and a 630 σ^B mutant.

Supplementary Tables

Table S1 Strains and plasmids used within this study

**Strains**	**Function**	**Origin**
E. coli		
DH5α	Cloning Vector	Invitrogen, USA
CA434	HB101(R702) Conjugation donor	(Purdy et al., 2002)
C. difficile		
R20291	Parental Strain	Trevor Lawley
Δ*rsbW*	R20291 with inframe deletion of *rsbW* from	This study
R20291 + pRPF185	WT Vector Control	This study
Δ*rsbW* + pRPF185	Mutant Vector Control	This study
Δ*rsbW*+*rsbW* (Δ*rsbW*+pRPF185::*rsbW*)	Complementation of rsbW with pRPF185rsbW	This study
Δ*spo0A*	Sporulation deficient mutant	Nigel Minton
**Plasmids**		
pMTL-SC7315	Allelic Exchange Vector	(Cartman et al., 2012)
pRPF185	Inducible expression system. Tetracycline-inducible gusA.	(Fagan & Fairweather, 2011)

Table S2

Oligonucleotides used within this study

Primer	Sequence (5’ → 3’)	Function
F1_*rsbW*	TTTTTTGTTTAAACTAAACAGCATAAATAAGGTTGT	Upstream flanking region with *PmeI*
R1_*rsbW*	GGGCTAGAATTCGTAATTTCCATCTTTATAGTCT	Upstream flanking region with *EcoRI*
F2_*rsbW*	GGGCTAGAATTCAATGACTAAATATTTAGGAGTTGA	Downstream flanking region with *EcoRI*
R2_*rsbW*	TTTTTTGTTTAAACTTGATATCCATAAGAAGCCTCC	Downstream flanking region with *PmeI*
SC7_F	GACGGATTTCACATTTGCCGTTTTGTAAACGAATTGCAGG	Sanger sequencing
SC7_R	AGATCCTTTGATCTTTTCTACGGGGTCTGACGCTCAGTGG	Sanger sequencing
Rsb_contr_F	TAGTAGGAAGCTCTGCTCTTATAGTAGC	Single crossover event
Rsb_contr_R	AATCTTCATACTCTATACTTCCAAAGTTACC	Single crossover event

Table S3. Generation Time of *C. difficile* strains grown in pH 4-7 (N=3)

	**Mean generation time (hrs) ± SD**			***p*-value**	
	**WT**	**Δ*rsbW***	**Δ*rsbW*+*rsbW***	**WT vs Δ*rsbW***	**Δ*rsbW*+*rsbW* vs Δ*rsbW***
**pH 4**	No Growth	No Growth	No Growth	N/A	N/A
**pH 5**	6.35 (± 0.85)	4.38 (± 0.11)	9.06 (± 1.76)	0.069	0.223
**pH 6**	3.06 (± 0.57)	3.31 (± 0.63)	4.28 (± 1.77)	0.739	0.530
**pH 7**	1.48 (± 0.38)	1.44 (± 0.04)	1.42 (± 0.57)	0.851	0.538

Table S5 Comparison of changes in gene expression in σ^B controlled genes between a Δ*rsbW* 20291 strain and a 630 σ^B mutant

	**R20291 (This study)**			**630 (Kint *et al.*, 2017)**		
**Function**	**Locus Tag**	**Log_2fold Change**	**P.adj**	**Locus Tag**	**Fold change**	**P-value**
**Oxidative and Nitrosative Stress**						
Rubreythrin	CDR20291_RS04325	-3.0525683	3.69E-06	CD0825	0.31	1.17E-02
Desulfoferrodoxin	CDR20291_RS04335	-3.0525683	3.69E-06	CD0827	0.18	0.00642
Oxidative stress glutamate synthase	CDR20291_RS04340	-2.4370593	0.0012576	CD0828	0.18	0.00642
NAD-specific glutamate synthase	CDR20291_RS01315	-3.0232748	0.000516	CD0179	0.36	7.00E-05
Reverse rubrerythrin (Rr)	CDR20291_RS07230	-1.4285605	0.0015016	CD1474	0.05	1.00E-05
CO dehydrogenase	CDR20291_RS01290	-2.3682142	5.24E-07	CD0174	0.01	0
Oxidoreductase, Fe-S subunit	CDR20291_RS01295	-2.0965647	4.61E-05	CD0175	0.02	0
Oxidoreductase, NAD/FAD binding subunit	CDR20291_RS01300	-2.1581865	7.02E-06	CD0176	0.02	0
NADH-oxidoreductase	CDR20291_RS08350	-1.4179498	0.0013855	CD1623	0.13	0
Anaerobic nitric oxide reductase	CDR20291_RS05575	-0.0251395	0.9728946	CD1157	0.05	0
Hydroxylamine reductase	CDR20291_RS11370	-1.4622148	0.0107004	CD2168	0.4	*1.84E-03
Nitroreductase-family protein	CDR20291_RS05380	-1.3269405	0.0215074	CD1125	0.06	0
Putative nitroreductase	CDR20291_RS04385	-0.2824522	0.6754431	CD0837	0.49	3.00E-04
Cysterine desulfurase family protein	CDR20291_RS19010	-0.4351186	0.3391618	CD3670	0.44	8.00E-05
Cysteine desulfurase	CDR20291_RS06220	-0.3321217	0.3847251	CD1279	0.16	0
Putative NifU-like protein	CDR20291_RS06225	-0.1515934	0.6943101	CD1280	0.16	0
Putative iron-sulfur assembly protein	CDR20291_RS18515	1.620109	1.69E-07	CD3607	0.5	0.002085
O-acetyl-serine thiol-lyase A	CDR20291_RS08200	0.1262661	0.8140441	CD1594	0.26	0.01311
Cyst(e)ine ABC transporter	CDR20291_RS11400	0.8150822	0.1403748	CD2174	0.43	0.00487
Cyst(e)ine ABC transporter	CDR20291_RS11410	0.7281516	0.1517188	CD2176	0.43	0.003375
Cyst(e)ine ABC transporter	CDR20291_RS11415	0.7521518	0.0927524	CD2177	0.48	0.002765
Sulfonate ABC transporter	CDR20291_RS07275	0.5338791	0.3193463	CD1482	0.32	0.00008
Sulfonate ABC transporter	CDR20291_RS07280	-1.4932156	0.0132286	CD1483	0.34	0.0001
Sulfonate ABC transporter	CDR20291_RS07285	-2.4079672	0.0005219	CD1484	0.14	5.00E-06

Table S5 Comparison of changes in gene expression in σ^B controlled genes between a Δ*rsbW* 20291 strain and a 630 σ^B mutant

Sulfonate ABC transporter	CDR20291_RS04830	-1.9643566	0.0009665	CD0999	0.27	0.00012
Methionine sulfoxide reducatase	CDR20291_RS11360	-1.1020349	0.0207907	CD2166	0.5	0.00152
Thioredoxin	CDR20291_RS08695	-0.9648861	0.0636023	CD1690	0.53	0.00676
Thiol-peroxidase	CDR20291_RS09365	0.3130606	0.4793811	CD1822	0.2	1.00E-05
Unknown function	CDR20291_RS09370	1.0843971	0.0090957	CD1823	0.3	0.00979
Nitrite and sulfite reductase subunit	CDR20291_RS09230	-4.2437332	0.0154	CD1796	0.46	1.30E-03
CoA disulfide reductase	CDR20291_RS09235	-4.0672454	0.0154833	CD1797	0.47	7.18E-03
**Other Stresses**						
ABC transporter, glycine betaine/carnitine/choline ATP binding protein	CDR20291_RS04720	-2.4869123	9.82E-09	CD0900	0.14	5.00E-06
ABC transporter, glycine betaine/carnitine/choline permease	CDR20291_RS04725	-2.3962506	2.01E-08	CD0901	0.19	0.00E+00
Glutamate 2,3-aminomutase	CDR20291_RS11760	-3.465794	1.01E-13	CD2252	0.36	9.50E-05
Excinuclease ABC subunit C	CDR20291_RS17380	-1.4032781	0.0017219	CD3410	0.43	1.51E-03
Excinuclease ABC subunit A	CDR20291_RS17385	-0.7777887	0.1416828	CD3411	0.35	1.20E-04
Excinuclease ABC subunit B	CDR20291_RS17390	-1.2872002	0.0077964	CD3412	0.29	6.00E-05
DNA mismatch repair protein MutS	CDR20291_RS10410	-0.089031	0.8742662	CD1977	0.37	0.00099
Tellurium resistance protein	CDR20291_RS08495	-0.2437041	0.7095546	CD1652	0.21	1.00E-05
Tellurium resistance protein	CDR20291_RS08405	-1.3033963	0.0044562	CD1634	0.47	2.60E-04
Tellurium resistance protein	CDR20291_RS08410	-1.2947752	0.0371161	CD1635	0.41	8.00E-05
Tellurium resistance protein	CDR20291_RS08415	-0.1678426	0.7427364	CD1636	0.63	5.76E-03
Tellurite associated resistance protein	CDR20291_RS08430	1.0109861	0.0113319	CD1639	0.52	1.99E-03
Glutaminase	CDR20291_RS02905	-0.4885554	0.4894025	CD0558	0.45	5.15E-04
Putative heavy-metal transport/detoxification protein	CDR20291_RS05450	1.2226782	0.0028179	CD1132	2.1	7.40E-04
**Heat shock protein, HSP33**	CDR20291_RS16590	0.5764517	0.2393333	CD3219	5.92	0
GroES protein	CDR20291_RS01380	0.5136971	0.447881	CD0193	1.88	1.00E-01

Table S5 Comparison of changes in gene expression in σ^B controlled genes between a Δ*rsbW* 20291 strain and a 630 σ^B mutant

**GroEL protein**	CDR20291_RS01385	-0.2572978	0.7129634	CD0194	1.8	2.42E-03
**Antibiotic Stress**						
**Two-component response regulator**	CDR20291_RS08355	-0.044644	0.9221157	CD1624	0.41	3.70E-04
**Two-component sensor histidine kinase**	CDR20291_RS08360	-0.1426602	0.7471562	CD1625	0.43	3.66E-03
**Transporter, Major Facilitator Superfamily**	CDR20291_RS13030	1.0737162	0.0119767	CD2506	0.45	1.28E-02
**Multidrug resistance Cme transporter**	CDR20291_RS16485	-0.9411767	0.041638	CD3198	2.46	7.69E-03
**ABC-transporter, multidrug-family**	CDR20291_RS14605	-0.2224725	0.5754239	CD2817	3.29	5.00E-05
**ABC-transporter, multidrug-family**	CDR20291_RS14610	0.3967146	0.2843091	CD2818	3.93	0.00015
**ABC-transporter, multidrug family ATP-binding protein**	CDR20291_RS01915	-1.2064601	0.0313504	CD0293	3.02	3.17E-03
**ABC-transporter, multidrug-family permease**	CDR20291_RS01920	-1.2466921	0.0720076	CD0294	2.73	0.00062
**ABC-transporter, multidrug-family**	CDR20291_RS07225	0.1313107	0.8198545	CD1473	2.5	6.95E-04
**Regulators**						
SinR	CDR20291_RS11605	5.2735733	1.30E-21	CD2214	2.03	0.0129
SinR’	CDR20291_RS11610	4.9035215	1.13E-19	CD2215	1.7	0.00966
**Spo0E**	CDR20291_RS16870	1.4960864	0.0026341	CD3271	0.11	1.00E-05
Spo0A	CDR20291_RS05865	0.3799281	0.3799281	CD1214	1.11	0.64
σ^E	CDR20291_RS13705	0.4847214	0.4481292	CD2643	7.82	0
σ^F	CDR20291_RS04065	1.6392501	0.0005497	CD0772	2.66	0.00132
σ^G	CDR20291_RS13700	-0.7293748	0.0940833	CD2642	12.13	0
σ^K	CDR20291_RS05945	0.6286375	0.1835021	CD1230	4.5	0
codY	CDR20291_RS06200	1.7667455	6.79E-05	CD1275	2.42	0.0004

Differentially expressed genes in **Bold** indicate discrepancies between R20291 (this study) and CD630 (Kint *et al.,* 2017).

References

Cartman, S. T., Kelly, M. L., Heeg, D., Heap, J. T., & Minton, N. P. (2012). Precise manipulation of the Clostridium difficile chromosome reveals a lack of association between the tcdC genotype and toxin production. *Applied and Environmental Microbiology*, *78*(13), 4683–4690. https://doi.org/10.1128/AEM.00249-12

Fagan, R. P., & Fairweather, N. F. (2011). Clostridium difficile has two parallel and essential sec secretion systems. *Journal of Biological Chemistry*, *286*(31), 27483–27493. https://doi.org/10.1074/jbc.M111.263889

Purdy, D., O'Keeffe, T. A. T., Elmore, M., Herbert, M., McLeod, A., Bokori-Brown, M., Ostrowski, A., & Minton, N. P. (2002). Conjugative transfer of clostridial shuttle vectors from Escherichia coli to Clostridium difficile through circumvention of the restriction barrier. *Molecular Microbiology*, *46*(2), 439–452. https://doi.org/10.1046/j.1365-2958.2002.03134.x

Figure S1

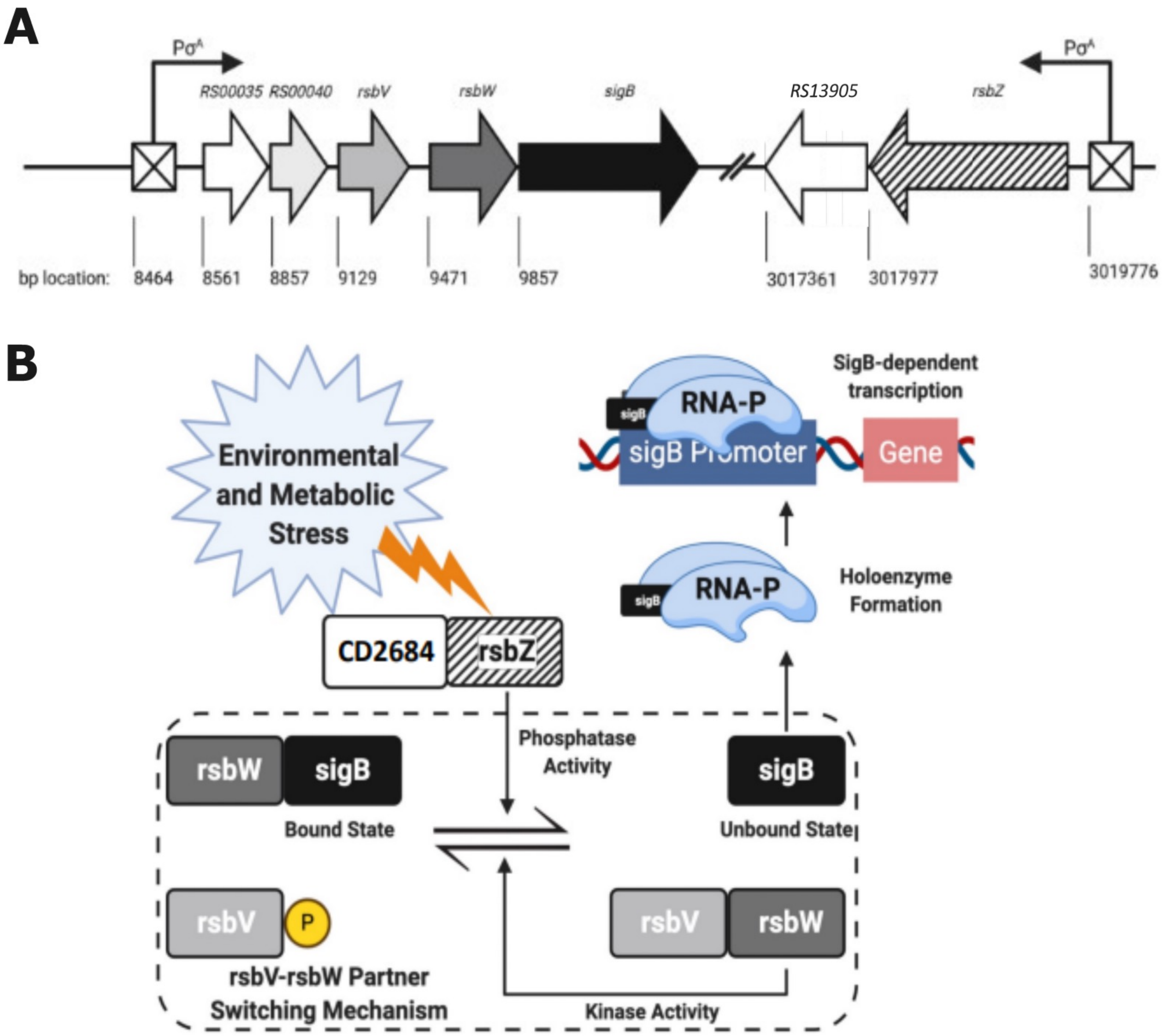
A
Pσ^A
RS00035
RS00040
rsbV
rsbW
sigB
RS13905
rsbZ
Pσ^A
bp location:
8464
8561
8857
9129
9471
9857
3017361
3017977
3019776
B
Environmental and Metabolic Stress
SigB-dependent transcription
RNA-P
sigB
sigB Promoter
Gene
Holoenzyme Formation
CD2684
rsbZ
Phosphatase Activity
rsbW
sigB
Bound State
sigB
Unbound State
rsbV
P
rsbV
rsbW
rsbV-rsbW Partner Switching Mechanism
Kinase Activity

Figure S2

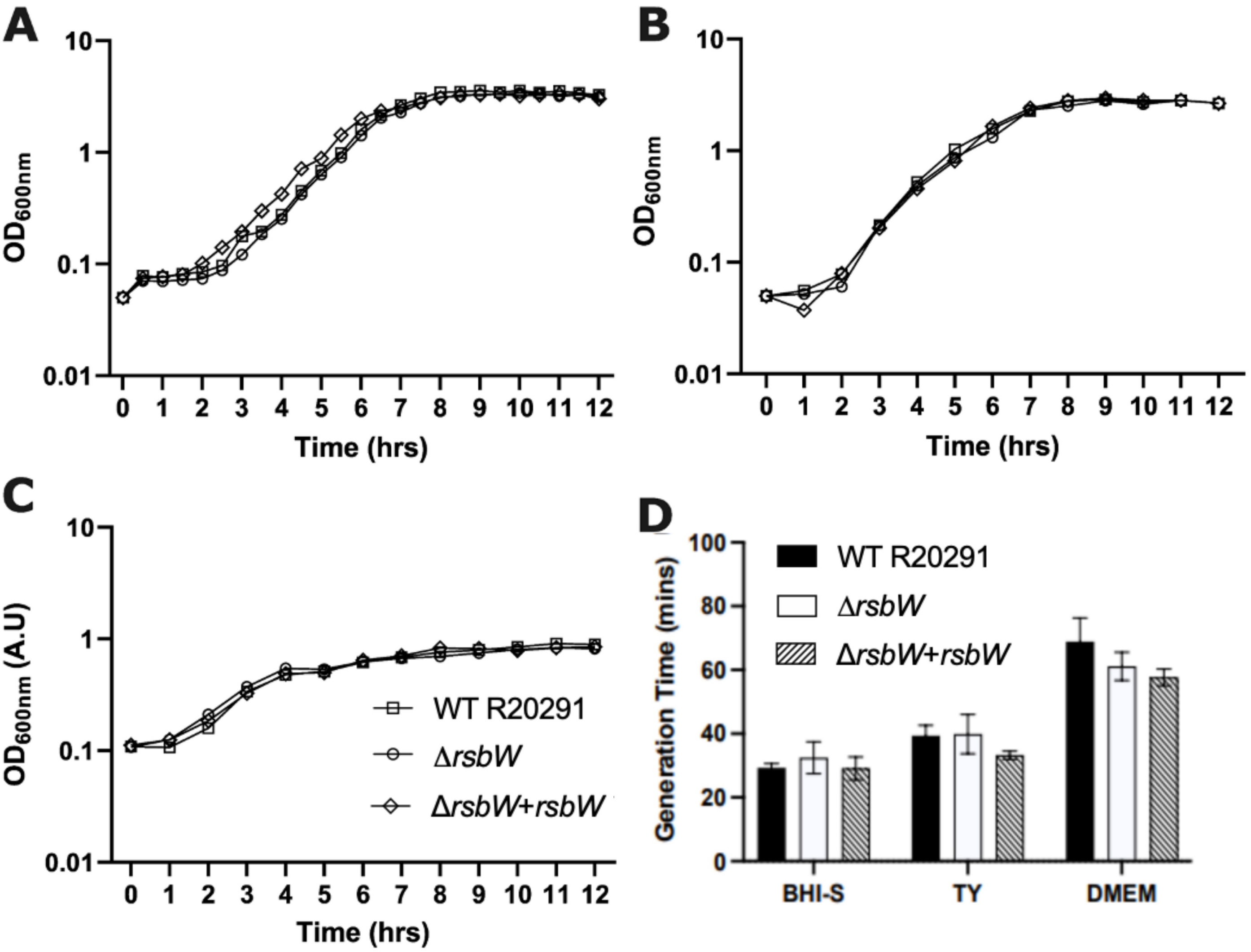
A
B
C
D
OD600nm
OD600nm (A.U)
Time (hrs)
10
1
0.1
0.01
0 1 2 3 4 5 6 7 8 9 10 11 12
WT R20291
ΔrsbW
ΔrsbW+rsbW
Generation Time (mins)
100
80
60
40
20
0
BHI-S
TY
DMEM

Figure S3

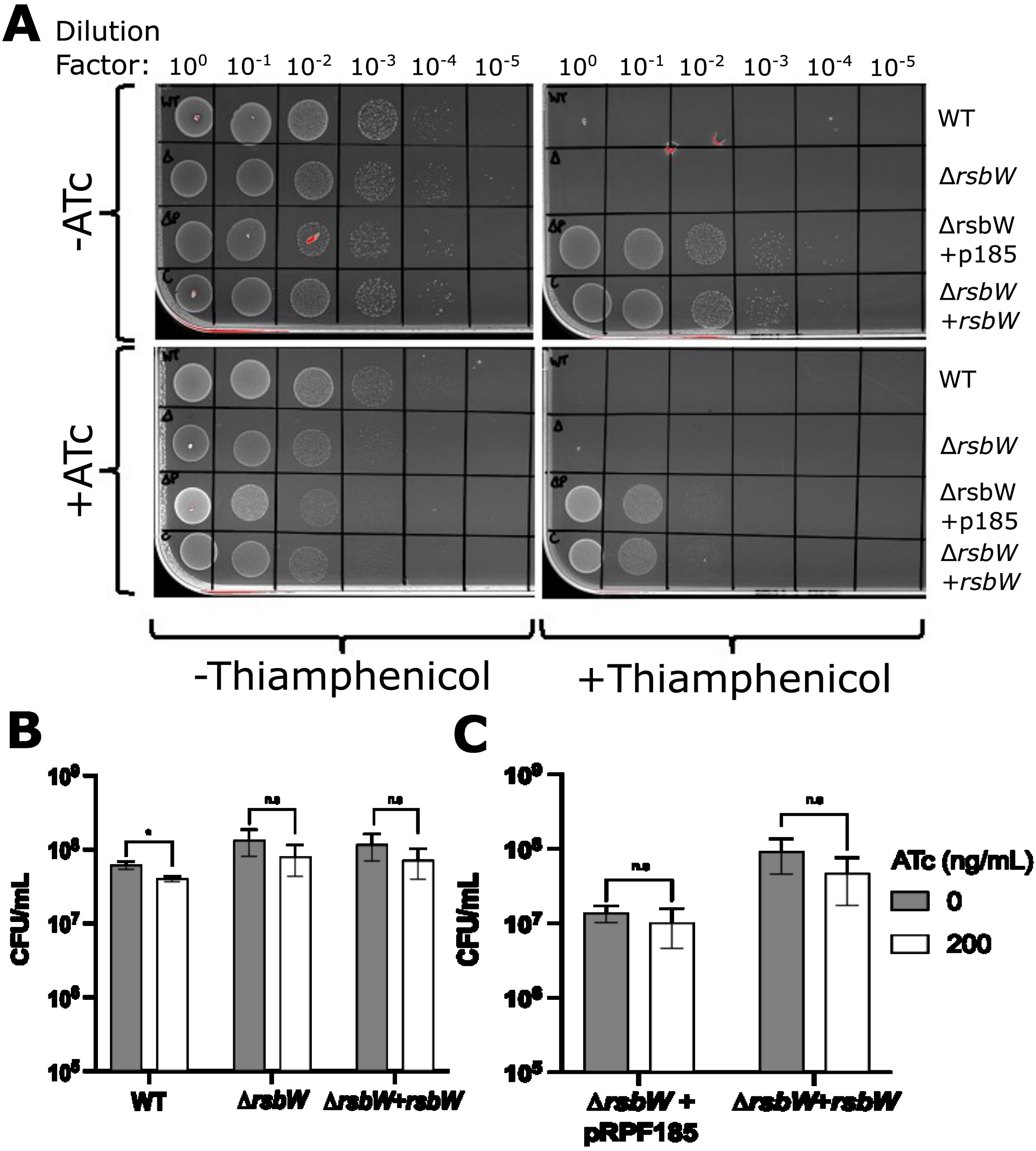

A
Dilution Factor: 10^0 10^-1 10^-2 10^-3 10^-4 10^-5
10^0 10^-1 10^-2 10^-3 10^-4 10^-5
-ATc
+ATc
WT
ΔrsbW
ΔrsbW +p185
ΔrsbW +rsbW
-Thiamphenicol
+Thiamphenicol
B
CFU/mL
10^9
10^8
10^7
10^6
10^5
*
n.s
WT
ΔrsbW
ΔrsbW+rsbW
C
ΔrsbW + pRPF185
ATc (ng/mL)
0
200

Figure S4

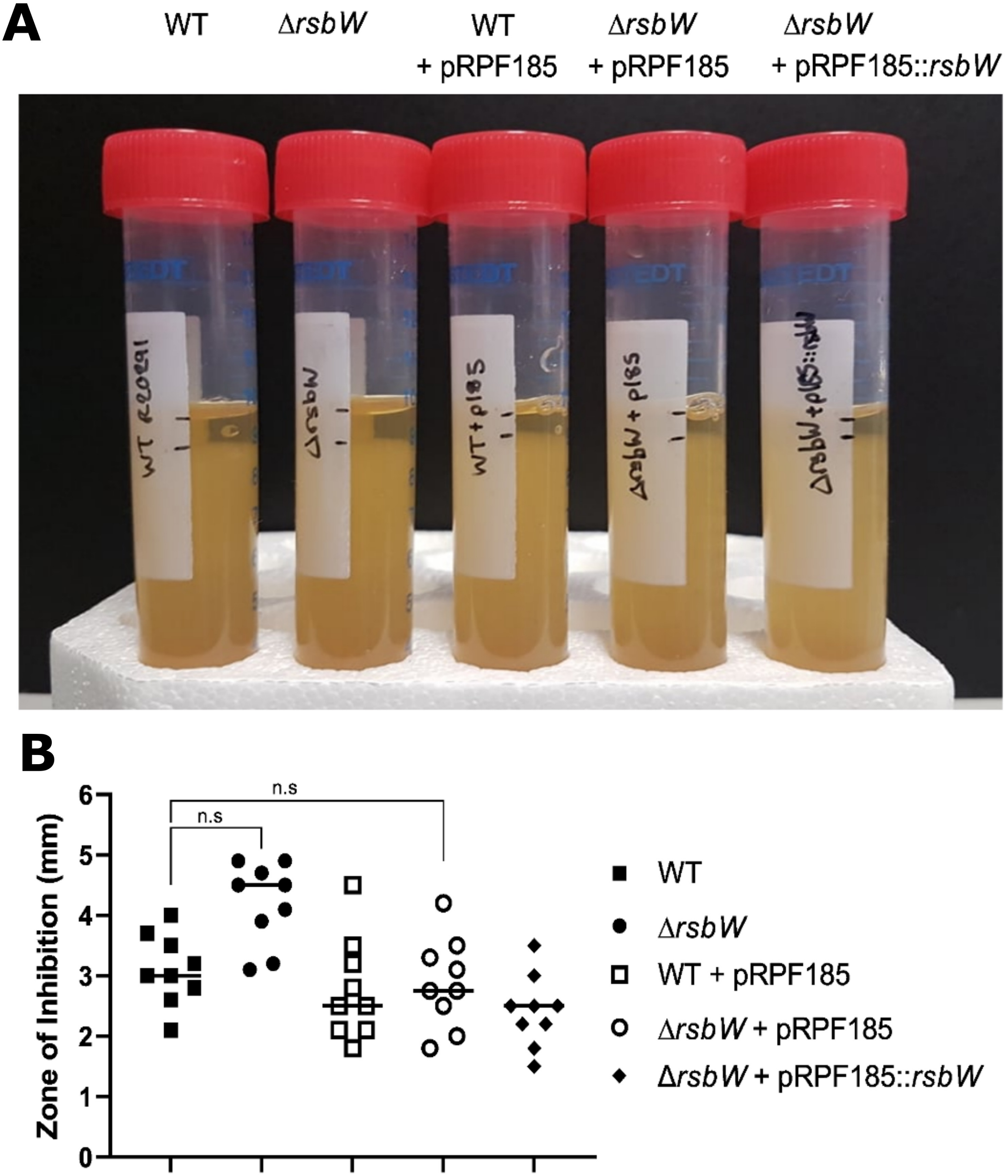

A
WT
ΔrsbW
WT + pRPF185
ΔrsbW + pRPF185
ΔrsbW + pRPF185::rsbW
B
Zone of Inhibition (mm)
0
1
2
3
4
5
6
n.s
n.s
WT
ΔrsbW
WT + pRPF185
ΔrsbW + pRPF185
ΔrsbW + pRPF185::rsbW

Figure S5

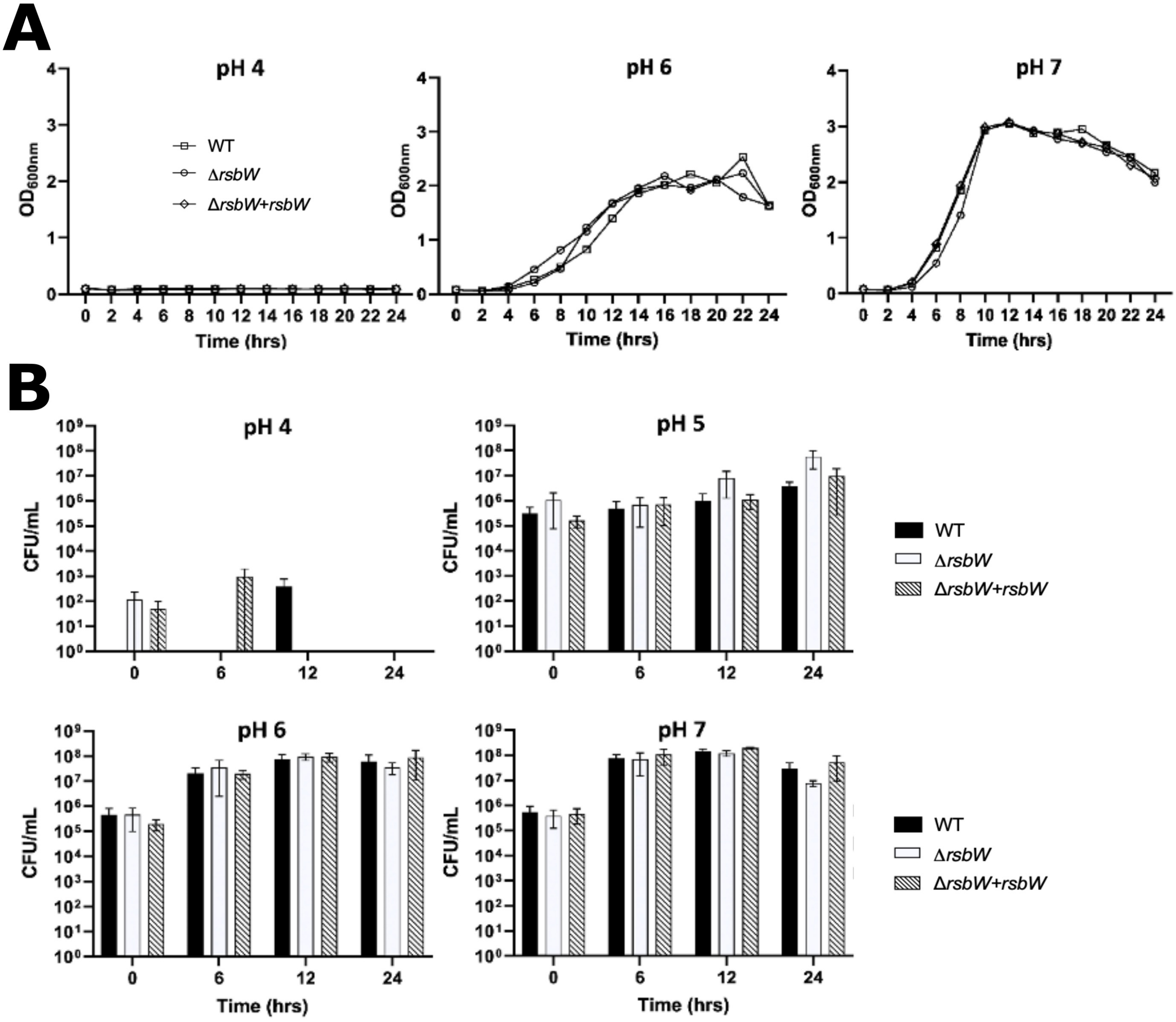
A
pH 4
pH 6
pH 7
OD600nm
Time (hrs)
WT
ΔrsbW
ΔrsbW+rsbW
B
pH 4
pH 5
pH 6
pH 7
CFU/mL
Time (hrs)
WT
ΔrsbW
ΔrsbW+rsbW

Figure S6

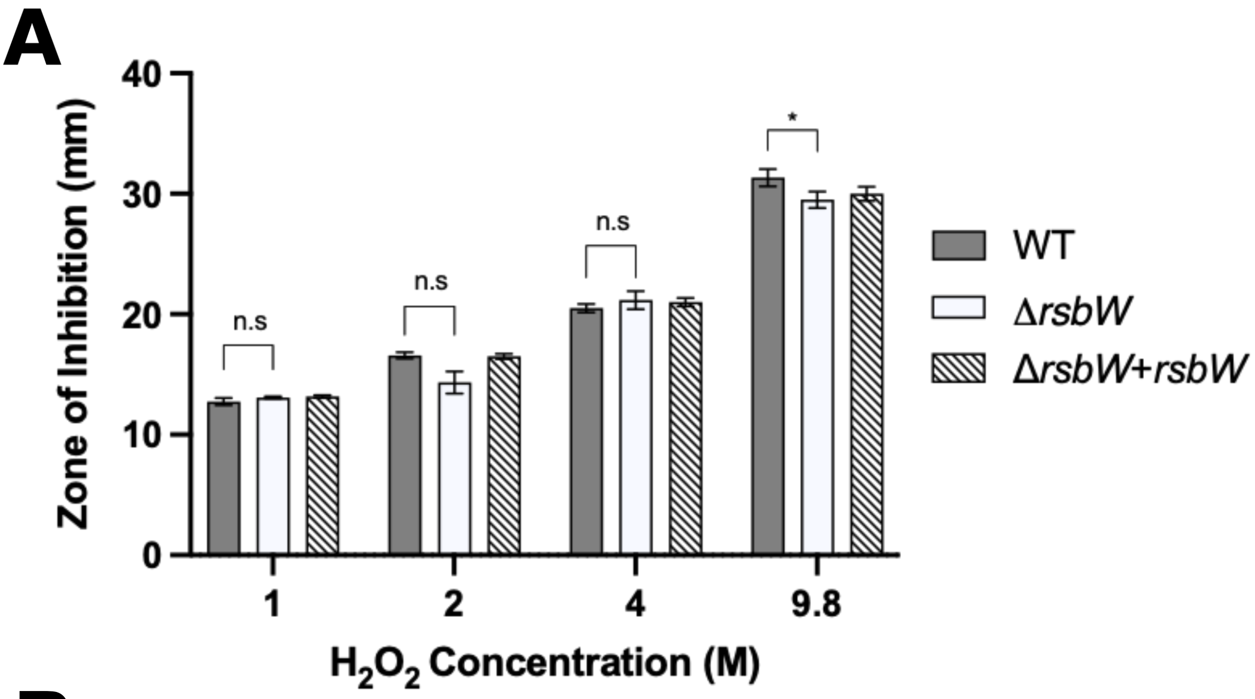
A
Zone of Inhibition (mm)
40
30
20
10
0
n.s
n.s
n.s
*
1
2
4
9.8
H_2O_2 Concentration (M)
WT
ΔrsbW
ΔrsbW+rsbW

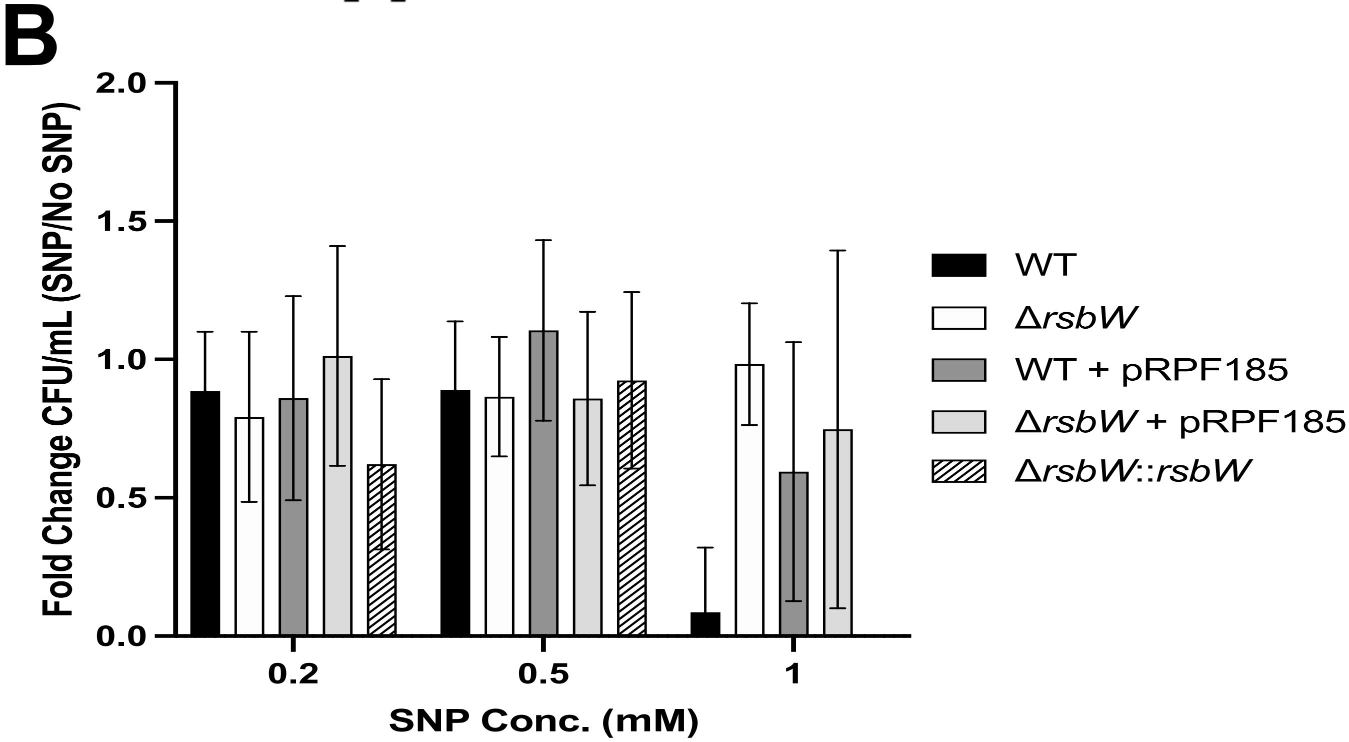
B
Fold Change CFU/mL (SNP/No SNP)
2.0
1.5
1.0
0.5
0.0
0.2
0.5
1
SNP Conc. (mM)
WT
ΔrsbW
WT + pRPF185
ΔrsbW + pRPF185
ΔrsbW::rsbW

Figure S7

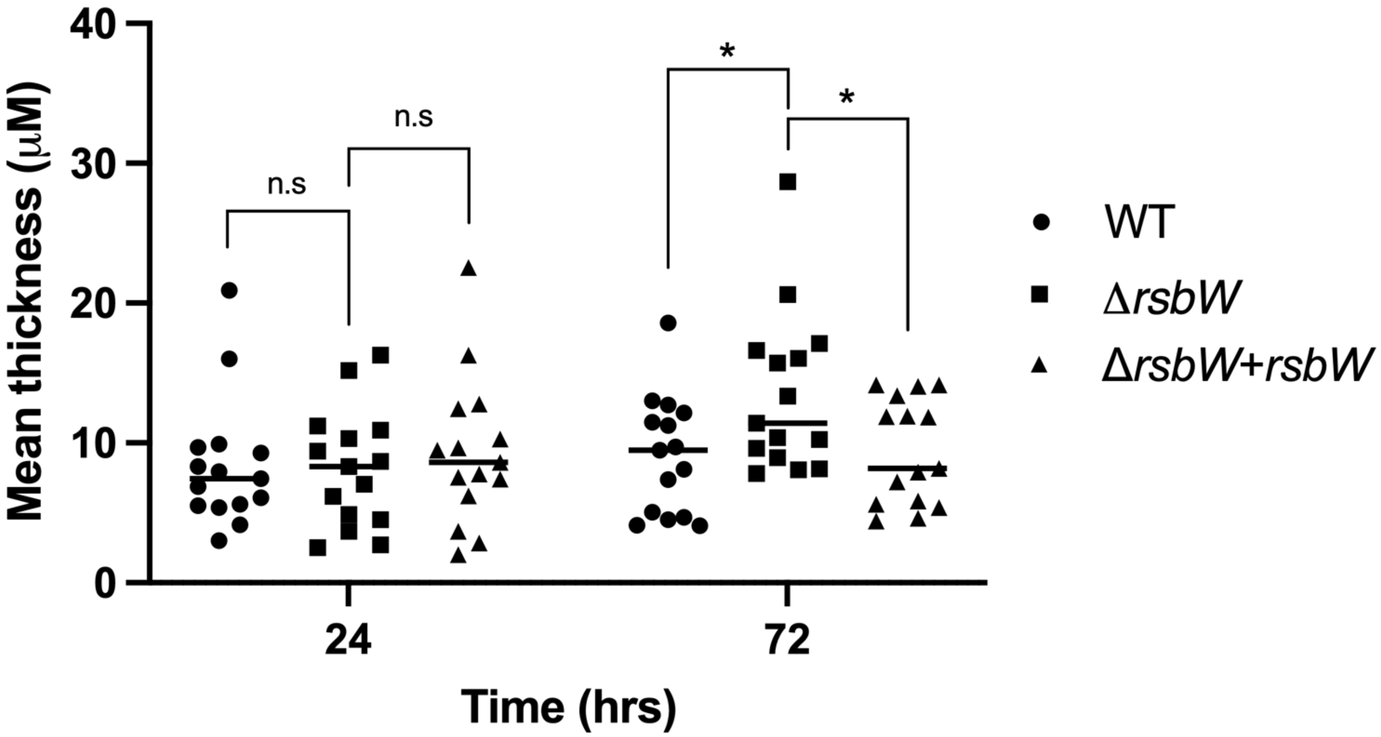

40
30
20
10
0
Mean thickness (μM)
n.s
n.s
*
*
24
72
Time (hrs)
WT
ΔrsbW
ΔrsbW+rsbW

Figure S8

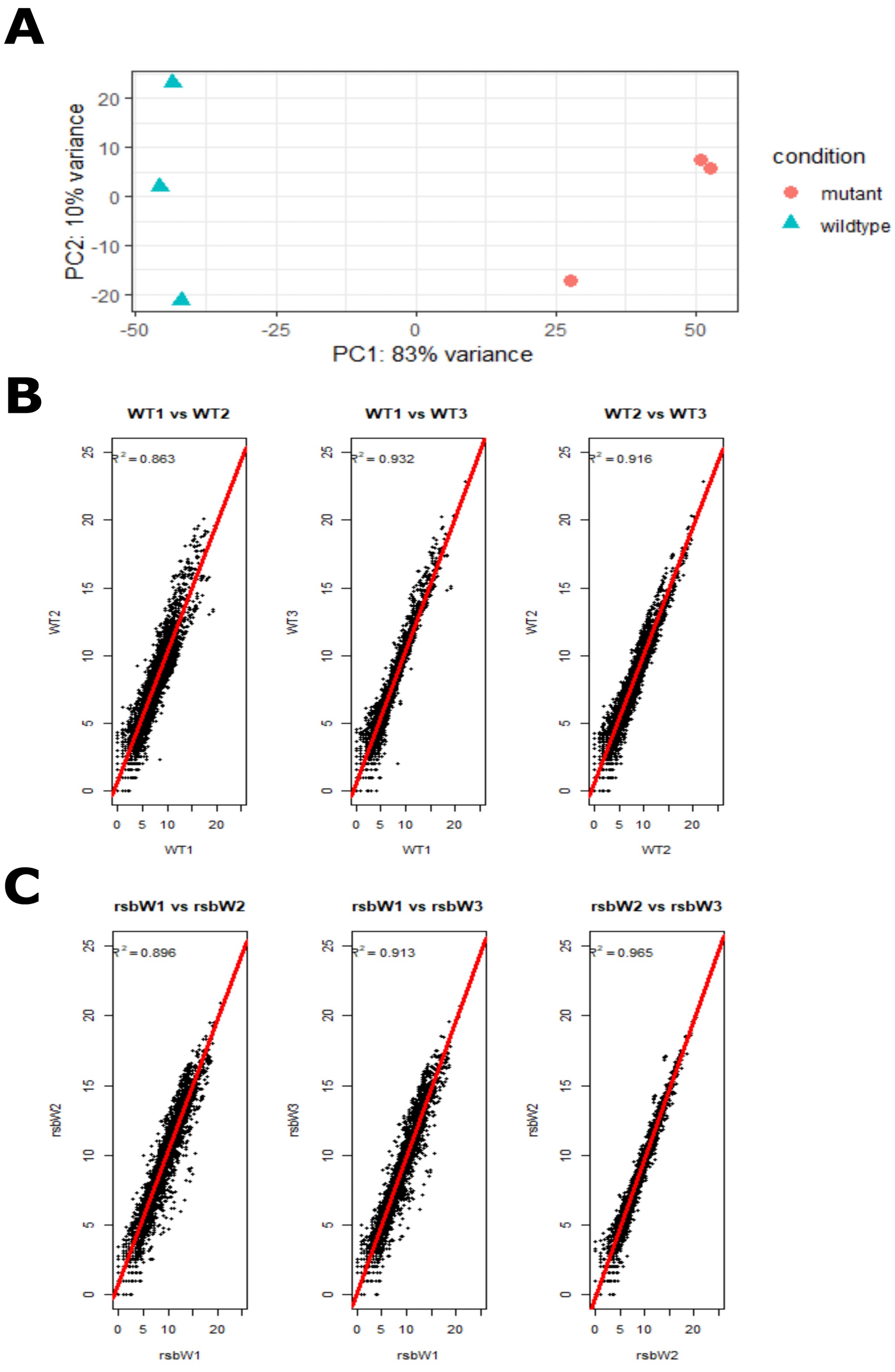
A
PC2: 10% variance
PC1: 83% variance
condition
mutant
wildtype
B
WT1 vs WT2
$R^2 = 0.863$
WT1 vs WT3
$R^2 = 0.932$
WT2 vs WT3
$R^2 = 0.916$
WT1
WT2
WT3
C
rsbW1 vs rsbW2
$R^2 = 0.896$
rsbW1 vs rsbW3
$R^2 = 0.913$
rsbW2 vs rsbW3
$R^2 = 0.965$
rsbW1
rsbW2
rsbW3

Figure S8

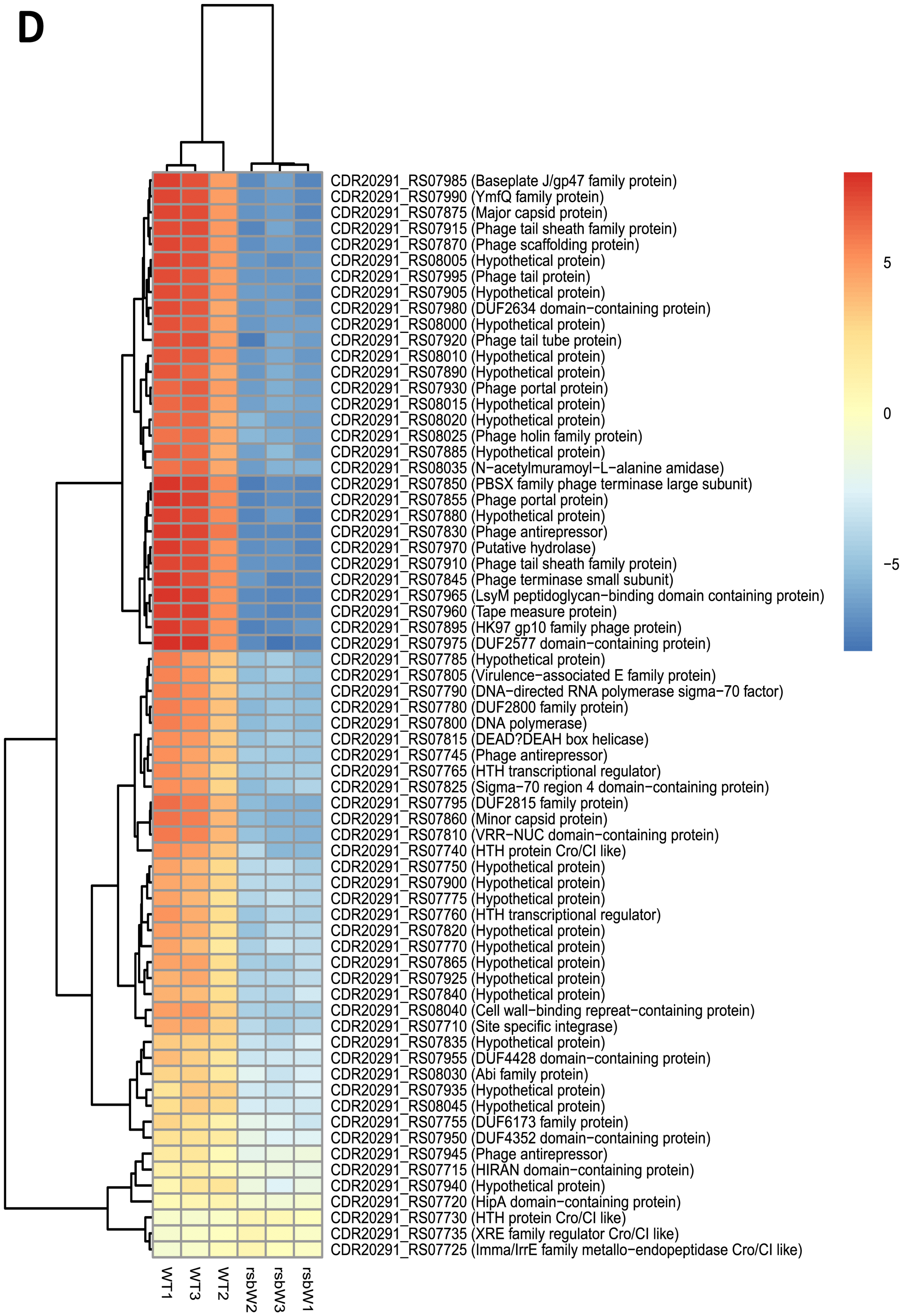
D
CDR20291_RS07985 (Baseplate J/gp47 family protein)
CDR20291_RS07990 (YmfQ family protein)
CDR20291_RS07875 (Major capsid protein)
CDR20291_RS07915 (Phage tail sheath family protein)
CDR20291_RS07870 (Phage scaffolding protein)
CDR20291_RS08005 (Hypothetical protein)
CDR20291_RS07995 (Phage tail protein)
CDR20291_RS07905 (Hypothetical protein)
CDR20291_RS07980 (DUF2634 domain−containing protein)
CDR20291_RS08000 (Hypothetical protein)
CDR20291_RS07920 (Phage tail tube protein)
CDR20291_RS08010 (Hypothetical protein)
CDR20291_RS07890 (Hypothetical protein)
CDR20291_RS07930 (Phage portal protein)
CDR20291_RS08015 (Hypothetical protein)
CDR20291_RS08020 (Hypothetical protein)
CDR20291_RS08025 (Phage holin family protein)
CDR20291_RS07885 (Hypothetical protein)
CDR20291_RS08035 (N−acetylmuramoyl−L−alanine amidase)
CDR20291_RS07850 (PBSX family phage terminase large subunit)
CDR20291_RS07855 (Phage portal protein)
CDR20291_RS07880 (Hypothetical protein)
CDR20291_RS07830 (Phage antirepressor)
CDR20291_RS07970 (Putative hydrolase)
CDR20291_RS07910 (Phage tail sheath family protein)
CDR20291_RS07845 (Phage terminase small subunit)
CDR20291_RS07965 (LsyM peptidoglycan−binding domain containing protein)
CDR20291_RS07960 (Tape measure protein)
CDR20291_RS07895 (HK97 gp10 family phage protein)
CDR20291_RS07975 (DUF2577 domain−containing protein)
CDR20291_RS07785 (Hypothetical protein)
CDR20291_RS07805 (Virulence−associated E family protein)
CDR20291_RS07790 (DNA−directed RNA polymerase sigma−70 factor)
CDR20291_RS07780 (DUF2800 family protein)
CDR20291_RS07800 (DNA polymerase)
CDR20291_RS07815 (DEAD?DEAH box helicase)
CDR20291_RS07745 (Phage antirepressor)
CDR20291_RS07765 (HTH transcriptional regulator)
CDR20291_RS07825 (Sigma−70 region 4 domain−containing protein)
CDR20291_RS07795 (DUF2815 family protein)
CDR20291_RS07860 (Minor capsid protein)
CDR20291_RS07810 (VRR−NUC domain−containing protein)
CDR20291_RS07740 (HTH protein Cro/CI like)
CDR20291_RS07750 (Hypothetical protein)
CDR20291_RS07900 (Hypothetical protein)
CDR20291_RS07775 (Hypothetical protein)
CDR20291_RS07760 (HTH transcriptional regulator)
CDR20291_RS07820 (Hypothetical protein)
CDR20291_RS07770 (Hypothetical protein)
CDR20291_RS07865 (Hypothetical protein)
CDR20291_RS07925 (Hypothetical protein)
CDR20291_RS07840 (Hypothetical protein)
CDR20291_RS08040 (Cell wall−binding repreat−containing protein)
CDR20291_RS07710 (Site specific integrase)
CDR20291_RS07835 (Hypothetical protein)
CDR20291_RS07955 (DUF4428 domain−containing protein)
CDR20291_RS08030 (Abi family protein)
CDR20291_RS07935 (Hypothetical protein)
CDR20291_RS08045 (Hypothetical protein)
CDR20291_RS07755 (DUF6173 family protein)
CDR20291_RS07950 (DUF4352 domain−containing protein)
CDR20291_RS07945 (Phage antirepressor)
CDR20291_RS07715 (HIRAN domain−containing protein)
CDR20291_RS07940 (Hypothetical protein)
CDR20291_RS07720 (HipA domain−containing protein)
CDR20291_RS07730 (HTH protein Cro/CI like)
CDR20291_RS07735 (XRE family regulator Cro/CI like)
CDR20291_RS07725 (Imma/IrrE family metallo−endopeptidase Cro/CI like)
WT1
WT3
WT2
rsbW2
rsbW3
rsbW1
5
0
−5

Figure S9

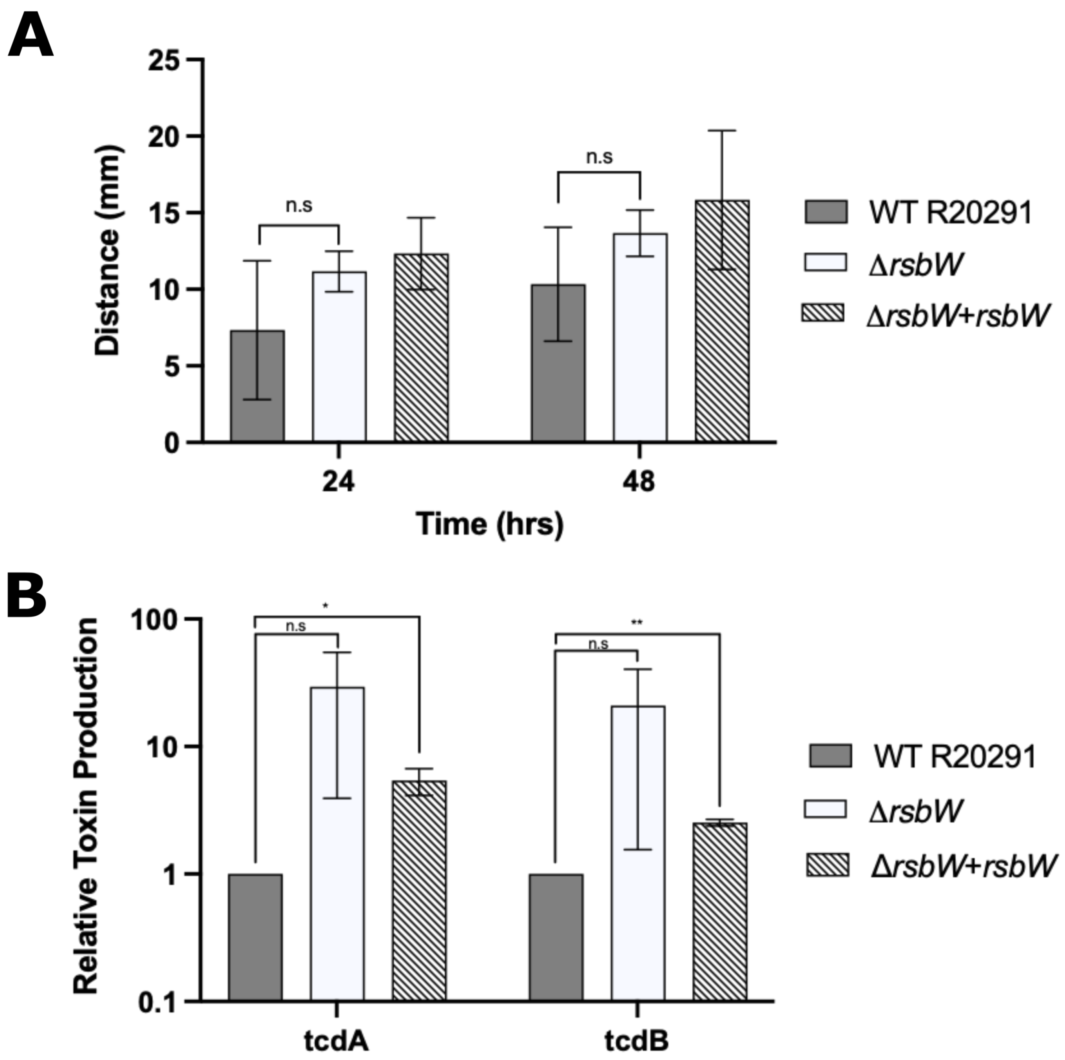
A
Distance (mm)
25
20
15
10
5
0
n.s
n.s
24
48
Time (hrs)
WT R20291
ΔrsbW
ΔrsbW+rsbW
B
Relative Toxin Production
100
10
1
0.1
*
n.s
**
n.s
tcdA
tcdB
WT R20291
ΔrsbW
ΔrsbW+rsbW

Figure S10

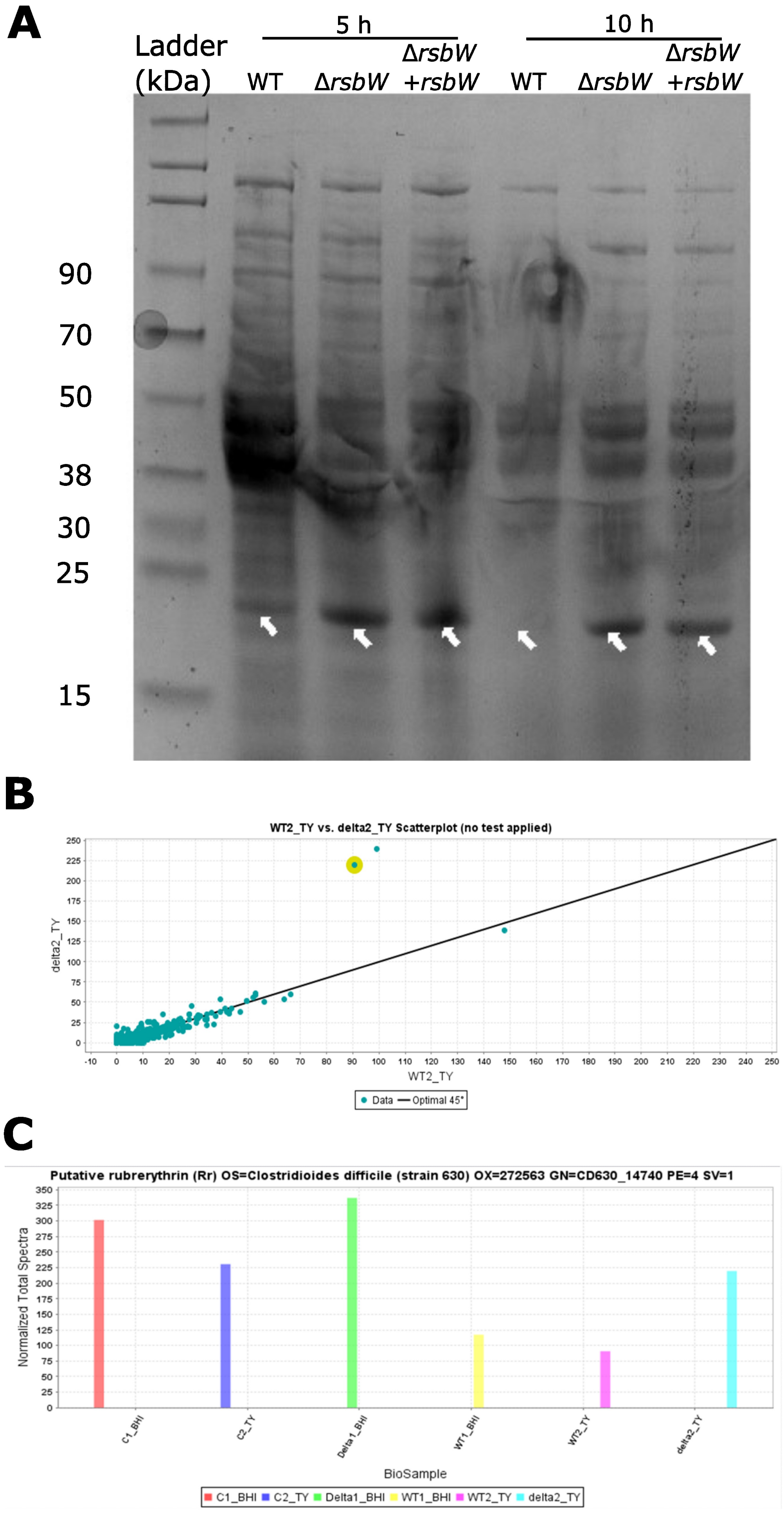
A
5 h
10 h
Ladder (kDa)
WT
ΔrsbW
ΔrsbW +rsbW
WT
ΔrsbW
ΔrsbW +rsbW
90
70
50
38
30
25
15
B
WT2_TY vs. delta2_TY Scatterplot (no test applied)
delta2_TY
WT2_TY
Data
Optimal 45°
C
Putative rubrerythrin (Rr) OS=Clostridioides difficile (strain 630) OX=272563 GN=CD630_14740 PE=4 SV=1
Normalized Total Spectra
BioSample
C1_BHI
C2_TY
Delta1_BHI
WT1_BHI
WT2_TY
delta2_TY
